# Plant Virus Genome Is Shaped by Specific Dinucleotide Restrictions That Influence Viral Infection

**DOI:** 10.1128/mBio.02818-19

**Published:** 2020-02-18

**Authors:** Alfonso González de Prádena, Adrián Sánchez Jimenez, David San León, Peter Simmonds, Juan Antonio García, Adrián A. Valli

**Affiliations:** a Centro Nacional de Biotecnología (CNB-CSIC), Madrid, Spain; b Leiden University Medical Center (LUMC), Leiden, The Netherlands; c Nuffield Department of Medicine, University of Oxford, Oxford, United Kingdom; CDC

**Keywords:** *Potyviridae*, potyvirus, RNA degradation, RNA virus, antiviral, plant defense, plant-microbe interactions, virus

## Abstract

The presence of CpG and UpA dinucleotides is restricted in the genomes of animal RNA viruses to avoid specific host defenses. We wondered whether a similar phenomenon exists in nonanimal RNA viruses. Here, we show that these two dinucleotides, especially UpA, are underrepresented in the family *Potyviridae*, the most important group of plant RNA viruses. Using plum pox virus (PPV; *Potyviridae* family) as a model, we show that an increase in UpA frequency strongly diminishes virus accumulation. Remarkably, unlike previous observations in animal viruses, PPV variants harboring CpG-rich fragments display just faint (or no) attenuation. The anticorrelation between UpA frequency and viral fitness additionally demonstrates the relevance of this particular dinucleotide: UpA-high mutants are attenuated in a dose-dependent manner, whereas a UpA-low variant displays better fitness than its parental control. Using high-throughput sequencing, we also show that UpA-rich PPV variants are genetically stable, without apparent changes in sequence that revert and/or compensate for the dinucleotide modification despite its attenuation. In addition, we also demonstrate here that the PPV restriction of UpA-rich variants works independently of the classical RNA silencing pathway. Finally, we show that the anticorrelation between UpA frequency and RNA accumulation applies to mRNA-like fragments produced by the host RNA polymerase II. Together, our results inform us about a dinucleotide-based system in plant cells that controls diverse RNAs, including RNA viruses.

## Abstract (precis)

Dinucleotides (combinations of two consecutive nucleotides) are not randomly present in RNA viruses; in fact, the presence of CpG and UpA is significantly repressed in their genomes. Although the meaning of this phenomenon remains obscure, recent studies with animal-infecting viruses have revealed that their low CpG/UpA frequency prevents virus restriction via a host antiviral system that recognizes, and promotes the degradation of, CpG/UpA-rich RNAs. Whether similar systems act in organisms from other life kingdoms has been unknown. To fill this gap in our knowledge, we built several synthetic variants of a plant RNA virus with deoptimized dinucleotide frequencies and analyzed their viral fitness and genome adaptation. In brief, our results inform us for the first time about an effective dinucleotide-based system that acts in plants against viruses. Remarkably, this viral restriction in plants is reminiscent of, but not identical to, the equivalent antiviral response in animals.

## INTRODUCTION

RNA viruses face fluctuating environments and are incredibly effective at adaptation when a selective pressure is applied (1). Central to this adaptive capacity is the enormous genetic diversity that characterizes RNA virus populations, which is mainly due to the distinctive low fidelity of the RNA-dependent RNA polymerases of these viruses and large population sizes (2). Therefore, with mutation rates varying between 10^−4^ and 10^−6^ errors per nucleotide, RNA virus infection produces a genetically heterogeneous population (quasispecies or mutant swarm or cloud) wherein individual RNA molecules differ from the consensus genome sequence at a few randomly mutated sites (3). Despite this flexibility, the genome sequences of RNA viruses are subject to poorly understood constraints observed through restrictions to certain synonymous mutations. Several reasons might account for these constraints in RNA virus genomes; apart from encoding viral proteins, virus genomic RNAs possess structural attributes, such as the formation of RNA secondary and tertiary structures relevant for interactions with both host and viral factors required for the different steps of viral infection (e.g., multiplication, movement) (4).

It has long been reported that the frequency of certain dinucleotides (two adjacent nucleotides in a linear sequence), particularly CpG and UpA, are pervasively suppressed in the genomes of many RNA viruses (5, 6). Such constraints have been hypothesized to be due to enzymatic modifications, dinucleotide stacking energies, and/or preferential mutations (5). Rapid progress in DNA synthesis over the last decade allowed the design and use of synthetic viruses, mainly infecting bacteria and animals (7–9). Along with this, advances in bioinformatics have accelerated experiments aiming to challenge hypotheses about restrictions in viral genomic sequences by the controlled introduction of a multitude of synonymous mutations at once. Indeed, these approaches have been very useful to identify *cis*-acting elements in viral genomes and further build deoptimized viruses with potential use in vaccination (10). Remarkably, these techniques have helped to unravel the long-standing mystery behind the suppression of CpG and UpA dinucleotides in animal viruses: boosting either of these two dinucleotides in viral genomes (without affecting codon usage) causes viral attenuation (11, 12), and this effect disappears when the expression of the animal, interferon-induced, zinc-finger antiviral protein (ZAP) or oligoadenylate synthetase (OAS3) is compromised (13, 14). Given that ZAP specifically interacts with CpG- and UpA-rich RNAs, the suppression of these dinucleotides in RNA virus genomes comprises a strategy that animal viruses developed to escape from the antiviral action of these proteins (13, 14).

Despite the importance of plant viruses, and the enormous negative consequences of viral diseases for sustainable agriculture and food security, much less is known about their genome restrictions at the level of dinucleotide composition. In fact, the low frequency of CpG and UpA in the genomes of plant-infecting RNA viruses was noticed a long time ago (5, 6) and reanalyzed more recently (15), but the biological meaning of this phenomenon is still unknown.

In this study, we manipulated the genome of plum pox virus (PPV) to find that an increased frequency of either CpG or, especially, UpA has a negative impact on viral fitness. A further comprehensive manipulation of UpA frequencies showed a dose-dependent effect, which extended to a mutant in which the UpA frequency was even lower than in the wild-type (WT) virus; such a mutant displayed greater fitness *in planta* than its parental control. Finally, aiming to understand the reasons underlying the UpA-based viral attenuation, we found that this restriction (i) is independent of the classic antiviral RNA silencing pathway, (ii) occurs at the level of RNA molecules, and (iii) also influences the accumulation of RNA polymerase II-transcribed RNAs.

## RESULTS

### Specific dinucleotide restrictions in the genome of potyvirids.

**FIG 1:**
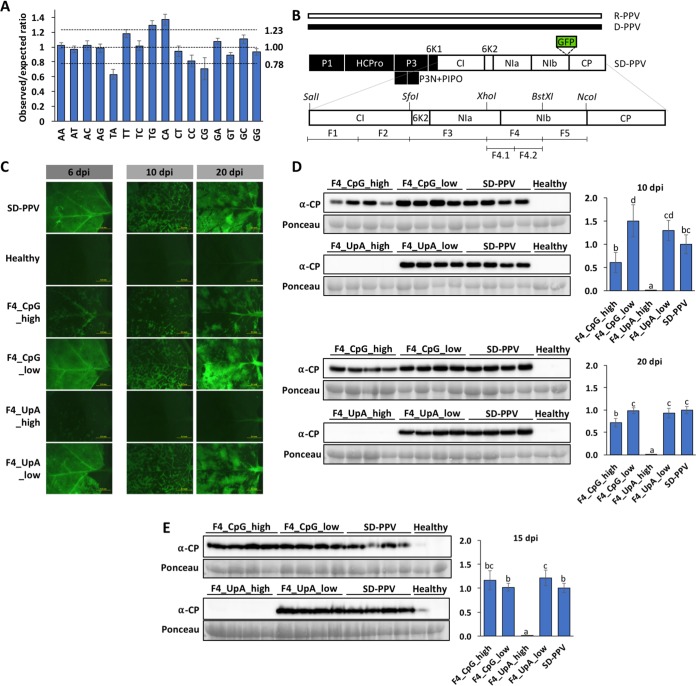
Functional analyses of CpG and UpA dinucleotide constraints in SD-PPV. (A) Histogram showing the mean ± SD of observed-to-expected (odds) ratios for different dinucleotides in potyvirid genomes. (B) Schematic representation of the SD-PPV chimera (each box represents a viral cistron) and its two parental viruses (PPV-R and PPV-D). Segments (F1 to F5) and restriction sites used to build the different SD-PPV derivatives are indicated. (C) Images taken with an epifluorescence microscope of leaves of N. benthamiana plants inoculated with the indicated mutant viruses (F4_) and their parental control (SD-PPV). Inoculated leaves at 6 days postinoculation (dpi) and upper noninoculated leaves at 10 and 20 dpi are shown. Bars, 5 mm. (D) PPV CP-specific immunoblot analyses of extracts from upper noninoculated N. benthamiana leaves (four individual plants) infected with the indicated mutant viruses (F4_) and their parental control (SD-PPV), at 10 and 20 dpi. (E) PPV CP-specific immunoblot analyses of extracts from upper noninoculated *P. persica* leaves (four individual plants) infected with the indicated viruses, at 15 dpi. Membranes stained with Ponceau red showing the RubisCO large subunit were included as loading controls. Bar graphs showing the mean ± SD (*n* = 4) of immunoblot signals in arbitrary units are shown on the right (for comparison, we considered the average for SD-PPV to be 1). Different letters indicate significant differences (*P* < 0.05), by one-way analysis of variance (ANOVA) and Tukey’s honestly significant difference (HSD) test.

The unprecedented amount of data provided by current sequencing technologies has greatly improved our knowledge about viruses and their genome sequences. This sequencing information allows a detailed compositional analysis of the genomes of members of the family *Potyviridae*, the most abundant and socioeconomically relevant group of plant RNA viruses. We followed the Karlin and Mrazek criteria to estimate over- and underrepresentation of the 16 dinucleotides (see Materials and Methods) (16) in 169 full-length potyvirid sequences deposited in NCBI to find the existence of an overall restriction for CpG and UpA dinucleotides (Fig. 1A; see Table S1 in the supplemental material). In contrast to animal RNA viruses (17), however, the average degree of suppression of UpA was significantly greater (*P* < 0.01, *t* test) and less variable than that of CpG (0.632 ± 0.066 for UpA versus 0.707 ± 0.146 for CpG) (Fig. 1A). The UpA odds ratio (OR) from 70% of the analyzed potyvirids was indeed lower than their corresponding CpG OR (Table S1).

10.1128/mBio.02818-19.5 TABLE S1 Odds ratio for members of the family *Potyviridae.* Download Table S1, PDF file, 0.1 MB. Copyright © 2020 González de Prádena et al. 2020 González de Prádena et al. This content is distributed under the terms of the Creative Commons Attribution 4.0 International license.

UpA and CpG dinucleotide restrictions are not an exclusive feature of potyvirid genomes, since equivalent analyses of other families of plant single-stranded (ss), positive-sense RNA viruses, such as *Alphaflexiviridae* (Table S2), *Closteroviridae* (Table S3), and *Bromoviridae* (Table S4), show the same dinucleotide bias. In fact, the preferential restriction of UpA over CpG is also observed in all these viruses; moreover, in the case of *Closteroviridae* and *Bromoviridae*, the presence of CpG is even greater than that of the cutoff-restricted dinucleotides (Tables S3 and S4). Finally, given that members of the *Bromoviridae* family are distinct from viruses belonging to the other mentioned families in regard to genome segmentation (tripartite versus monopartite), size of the RNA molecules (small versus large), and virus particle structure (icosahedral versus flexuous rod), we envisage that the observed dinucleotide bias is a general feature of plant positive-strand RNA viruses.

10.1128/mBio.02818-19.6 TABLE S2 Odds ratio for members of the family *Alphaflexiviridae.* Download Table S2, PDF file, 0.1 MB. Copyright © 2020 González de Prádena et al. 2020 González de Prádena et al. This content is distributed under the terms of the Creative Commons Attribution 4.0 International license.

10.1128/mBio.02818-19.7 TABLE S3 Odds ratio for members of the family *Closteroviridae.* Download Table S3, PDF file, 0.1 MB. Copyright © 2020 González de Prádena et al. 2020 González de Prádena et al. This content is distributed under the terms of the Creative Commons Attribution 4.0 International license.

10.1128/mBio.02818-19.8 TABLE S4 Odds ratio for members of the family *Bromoviridae*. Download Table S4, PDF file, 0.1 MB. Copyright © 2020 González de Prádena et al. 2020 González de Prádena et al. This content is distributed under the terms of the Creative Commons Attribution 4.0 International license.

### Absence of essential *cis*-acting RNA elements in the central part of the PPV genome.

We selected PPV (*Potyviridae* family, *Potyvirus* genus) (Fig. 1B) as a model to study the importance of dinucleotide restrictions in plant RNA viruses because it showed degrees of CpG and UpA suppression that is typical of members of this RNA virus family and because its genome can be experimentally manipulated in a previously established reverse genetics model (18). First, we defined regions in the PPV genome harboring neither expected relevant RNA secondary structures nor overlapping open reading frames (ORFs) to isolate the effects of dinucleotide manipulation from other causes of attenuation. To identify these, PPV genomes were aligned, and the coding regions were analyzed for synonymous variability and predicted RNA structure formation (Fig. S1A). Based on its variability and folding energy, a region between positions 3633 and 9296 was identified as suitable for the intended mutagenesis.

10.1128/mBio.02818-19.1 FIG S1 Scanning the PPV genome to identify target regions suitable for manipulation of dinucleotide frequencies. (A) Scan of synonymous variability (red line; left-hand axis scale) among 61 aligned nonredundant (>1% divergence) PPV sequences to identify regions of suppressed variability indicative of overlapping reading frames and structured RNA elements. A plot of MFED (blue line; right-hand *y*-axis scale) has been superimposed to identify areas of thermodynamically favored base pairing. (B) Images taken with an epifluorescence microscope of upper noninoculated leaves, at 10 and 20 dpi, from plants infected with the indicated permuted viruses (_perm) and their parental control (SD-PPV). Bars, 5 mm. (C and D) PPV CP-specific immunoblot analyses of extracts from upper noninoculated N. benthamiana leaves (four individual plants) infected with the indicated viruses at 10 (C) and 20 (D) dpi. Membranes stained with Ponceau red showing the RubisCO large subunit were included as loading controls. Bar graphs showing the mean ± SD (*n* = 4) of immunoblot signals in arbitrary units are shown on the right (for comparison, we considered the average for SD-PPV to be 1). Different letters indicate significant differences (*P* < 0.05), by one-way ANOVA and Tukey’s HSD test. Download FIG S1, JPG file, 0.4 MB. Copyright © 2020 González de Prádena et al. 2020 González de Prádena et al. This content is distributed under the terms of the Creative Commons Attribution 4.0 International license.

In order to validate this prediction, we built PPV permuted mutants in which sequences were maximally scrambled while keeping coding information and native mono- and dinucleotide frequencies identical to the wild-type sequence (algorithm CDLR). It is worth mentioning here that these modifications, as well as those introduced to change dinucleotide frequencies (see below), did not significantly alter codon usage (see the codon adaptation index [CAI] column in Table S5), so that potential problems with translation rate are minimized. The pLX-SD-PPV infectious clone, which expresses the SD-PPV chimera (see Materials and Methods), and five derivative CDLR mutants (F1_perm, F2_perm, F3_perm, F4_perm, and F5_perm) were agroinoculated in Nicotiana benthamiana plants, and the infection was followed under UV light. All five SD-PPV permuted derivatives showed infection kinetics similar to that of the parental virus, with comparable fluorescence in upper noninoculated leaves at 10 and 20 days postinoculation (dpi) (Fig. S1B). Consistent with green fluorescent protein (GFP) detection, similar accumulations of these viruses were observed in upper noninoculated leaves by quantitation of PPV capsid protein (CP) by immunoblotting (Fig. S1C and D). These observations support the idea that the central region of the PPV genome does not harbor relevant overlapping ORFs or *cis*-acting RNA elements; hence, any of these five segments may be potentially manipulated to determine the effects of CpG and UpA dinucleotide frequencies on PPV infection.

10.1128/mBio.02818-19.9 TABLE S5 Composition of different PPV-derived fragments. Download Table S5, PDF file, 0.1 MB. Copyright © 2020 González de Prádena et al. 2020 González de Prádena et al. This content is distributed under the terms of the Creative Commons Attribution 4.0 International license.

### Attenuation of viruses with deoptimized dinucleotide frequencies.

First, we evaluated the effect of CpG and UpA dinucleotide compositional modification of segment F4 in mutants, using agroinfiltration as the inoculation method (Fig. 1B) (see Materials and Methods for details). Leaves agroinoculated with SD-PPV mutants in which the number of CpG or UpA dinucleotides had been reduced (F4_CpG_low and F4_UpA_low) displayed strong fluorescence signals at 6 dpi, comparable to, or greater than, that of WT virus. Contrastingly, leaves agroinoculated with F4_CpG_high and F4_UpA_high showed reduced fluorescence, almost undetectable in the case of the F4_UpA_high mutant (Fig. 1C). At 10 and 20 dpi, GFP was clearly detected in upper noninoculated leaves of plants inoculated with the SD-PPV positive control, F4_CpG_low, and F4_UpA_low and, at a lower level, in those from F4_CpG_high-infected plants (Fig. 1C). Strikingly, plants inoculated with F4_UpA_high showed no fluorescence in these tissues, even at 20 dpi (Fig. 1C). Estimations of viral accumulation in upper noninoculated leaves by immunoblotting (Fig. 1D) were consistent with the fluorescence observations.

The reduced ability of F4_UpA_high to move systemically was also observed when its corresponding infectious cDNA clone was delivered by biolistics instead of agroinoculation (data not shown), suggesting that attenuation induced by dinucleotide frequency changes was independent of the inoculation method. We also showed that some aspects of the attenuation may be host dependent. The SD-PPV chimera can infect *Prunus* trees. We agroinoculated Prunus persica, a natural host of PPV, with SD-PPV and derivative viruses having altered dinucleotide frequencies in F4. Analogously to N. benthamiana plants, the F4_UpA_high mutant was unable to reach upper noninoculated leaves, whereas SD-PPV and the remaining mutants accumulated at comparable levels in these tissues, as observed by immunoblotting at 15 dpi (Fig. 1E). However, in contrast to infections in N. benthamiana, we observed no differences in CP accumulation between F4_CpG_high and SD-PPV in *P. persica* (Fig. 1E); hence, CpG-induced attenuation may represent the outcome of interactions with host factors other than those interacting with UpA-high mutants.

A similar pattern of attenuation of F4 mutants in N. benthamiana was observed in mutants with equivalently mutagenized sequences in the F3 region, a different 1-kb fragment of SD-PPV (Fig. 1B). An increase in UpA frequency in F3 produced comparable attenuation, with the F3_UpA_high mutant being undetectable by both fluorescence and immunoblotting in upper noninoculated leaves of infected N. benthamiana plants (Fig. S2E and F). The F3_CpG_high mutant showed a reduced degree of attenuation, even lower than the restriction observed in the equivalent F4 mutant (Fig. S2A and B). No differences were observed in viral accumulation in plants infected with SD-PPV, F3_CpG_low, or F3_UpA_low (Fig. S2A to D). For both regions, the fitness ranking of mutants in N. benthamiana was as follows: SD-PPV ≃ CpG_low ≃ UpA_low > CpG_high ≫ UpA_high.

10.1128/mBio.02818-19.2 FIG S2 Functional analysis of CpG and UpA dinucleotide constraints in another segment of the SD-PPV genome. (A, C, and E) Images taken with an epifluorescence microscope of N. benthamiana leaves infected with the indicated mutant viruses (F3_) and their parental control (SD-PPV). Inoculated leaves at 6 days postinoculation (dpi) and upper noninoculated leaves at 10 and 20 dpi are shown. Bars, 5 mm. (B, D, and F) PPV CP-specific immunoblot analyses of extracts from upper noninoculated N. benthamiana leaves (four individual plants) infected with the indicated mutant viruses (F3_) and their parental control (SD-PPV) at 10 and 20 dpi. Membranes stained with Ponceau red showing the RubisCO large subunit were included as loading controls. Bar graphs showing the mean ± SD (*n* = 4) of immunoblot signals in arbitrary units are shown on the right (for comparison, we considered the average for SD-PPV to be 1). Different letters indicate significant differences (*P* < 0.05), by one-way ANOVA and Tukey’s HSD test. For this experiment, all viruses were inoculated by agroinfiltration, with the exception of F3_UpA_high, whose infectious cDNA plasmid was unable to transform Agrobacterium tumefaciens due to unknown reasons. Hence, the inoculation of this particular mutant and its parental control was carried out by biolistics in an independent experiment (E and F). Download FIG S2, JPG file, 0.4 MB. Copyright © 2020 González de Prádena et al. 2020 González de Prádena et al. This content is distributed under the terms of the Creative Commons Attribution 4.0 International license.

From the experiments presented so far, we conclude that the increase of either CpG or UpA frequencies in two different segments of the SD-PPV genome results in modest and strong virus attenuation, respectively. The difference in degree of attenuation observed between UpA_high and CpG_high mutant viruses correlates positively with the greater restriction for UpAs observed in potyvirid genome sequences (see above). For this reason, we decided to concentrate further efforts on understanding the relevance of this particular dinucleotide for viral fitness.

### The increase of UpAs, but neither that of ApUs nor U+A mononucleotides, attenuates viral infection.

**FIG 2:**
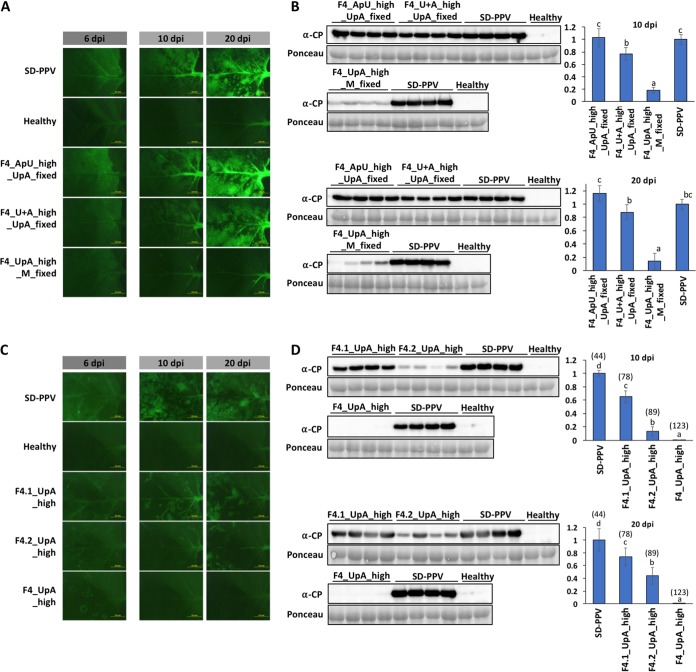
Influence of UpA dinucleotide and U+A mononucleotide contents on SD-PPV fitness. (A and C) Images taken with an epifluorescence microscope of N. benthamiana leaves infected with the indicated viruses. Inoculated leaves at 6 days postinoculation (dpi) and upper noninoculated leaves at 10 and 20 dpi are shown. Bars, 5 mm. (B and D) PPV CP-specific immunoblot analyses of extracts from upper noninoculated N. benthamiana leaves (four individual plants) infected with the indicated mutant viruses (F4_) and their parental control (SD-PPV), at 10 (two upper membranes) and 20 (two lower membranes) dpi. Membranes stained with Ponceau red showing the RubisCO large subunit were included as loading controls. Bar graphs showing the mean ± SD (*n* = 4) of immunoblot signals in arbitrary units are shown on the right (for comparison, we considered the average for SD-PPV to be 1). In panel D, the number of UpAs in the whole fragment F4 from each virus is indicated in parentheses above its corresponding bar. Different letters indicate significant differences (*P* < 0.05), by one-way ANOVA and Tukey’s HSD test.

The frequency of both U and A mononucleotides was considerably increased in the F4_UpA_high mutant as a consequence of increasing UpA frequencies (Table S5). To rule out the possibility that the attenuation of this virus resulted from the elevated, and potentially nonphysiological, frequencies of U and A mononucleotides rather than UpA dinucleotides, we built three additional SD-PPV-derived viruses with mutations in F4: (i) F4_UpA_high_M_fixed, in which the number of UpAs in F4 was increased, but to a lower extent than that in the F4_UpA_high mutant, while the frequency of U and A mononucleotides was kept identical to the WT sequence; (ii) F4_U+A_high_UpA_fixed, in which U and A mononucleotides in F4 were increased to levels of the original F4_UpA_high mutant but the frequency of UpA was kept identical to that of the WT; and (iii) F4_ApU_high_UpA_fixed, in which the number of ApUs in F4 was increased to match the number of UpAs in F4_UpA_high while the numbers of UpA were kept the same as in the WT. As for the other mutants, all sequences encoded the same proteins (Table S5). We agroinoculated N. benthamiana plants with these mutants and SD-PPV as the control, and infections were followed under UV light. Although all mutants displayed similar levels of fluorescence in inoculated leaves at 6 dpi (Fig. 2A), F4_UpA_high_M_fixed was substantially attenuated, with much fainter fluorescence in upper noninoculated tissues at both 10 and 20 dpi (Fig. 2A). Detection of CP by immunoblotting confirmed the attenuation of F4_UpA_high_M_fixed (Fig. 2B). Increasing UpA frequencies while keeping U+A mononucleotide frequencies constant therefore still led to attenuation. In contrast, F4_U+A_high_UpA_fixed showed only minimally reduced accumulation compared to the parental control (Fig. 2B), similarly discounting a significant role of high U and A mononucleotide frequencies in causing attenuation (Fig. 1C to E). Finally, the ApU-high mutant accumulated identically to SD-PPV (Fig. 2A and B). These control experiments clearly demonstrate that UpA dinucleotide frequencies, and not other combinations of U and A, were responsible for the observed attenuation phenotypes.

### Dose-dependent attenuation of UpA-rich viruses.

In designing the F4_UpA_high_M_fixed sequences, keeping mononucleotide frequencies constant prevented sequences from being generated with all 123 UpA dinucleotides, as in the original UpA-high mutant. The maximum achievable final number was 104 instead of 123 (compared to 44 in the wild-type sequence), and this may have contributed to the noticeably lower degree of attenuation. To investigate the dose dependence of UpA addition on viral fitness, we divided the F4_UpA_high sequence into two halves, F4.1 and F4.2 (Fig. 1B), containing 58 UpAs and 65 UpAs (compared to 24 and 20 in the wild-type sequence), respectively. New mutants in which the mutated regions were exchanged with the corresponding wild-type sequences were created to produce F4.1_UpA_high and F4.2_UpA_high mutants (Table S5). If attenuation was dose dependent, then the predicted fitness ranking would be as follows: SD-PPV ≃ F4_ApU_high > F4.1_UpA_high > F4.2_UpA_high > F4_UpA_high_M_fixed > F4_UpA_high. Through observation of fluorescence in both agroinoculated and upper noninoculated leaves of N. benthamiana (Fig. 2A and C), as well as estimation of virus loads in systemically infected tissues by immunoblotting (Fig. 2B and D), this fitness ranking was precisely reproduced, supporting the idea that virus fitness is inversely proportional to the UpA frequency in the viral genomic RNA.

### Improved fitness of a mutant virus with lower UpA frequency.

**FIG 3:**
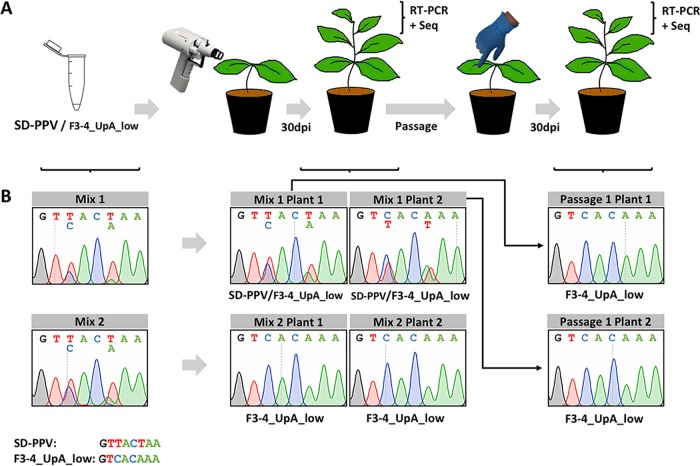
Competition between a low-UpA virus and its parental control. (A) Schematic representation of the competition experiment. Microcarrier cartridges were prepared with gold particles coated with a DNA mixture of the indicated plasmids and used to inoculate N. benthamiana plants by shooting. Thirty days postinoculation (dpi), upper noninoculated leaves were collected and used to determine the composition of the viral progeny. Extracts from infected plants in which competitors accumulated at similar levels were used to inoculate new N. benthamiana plants, whose viral progeny was also analyzed at 30 dpi in upper noninoculated leaves. (B) Images showing a representative region of the chromatogram obtained from the sequencing of PCR products from inoculated DNA mixes and viral progeny cDNAs. This region includes two single-nucleotide polymorphisms (SNPs) in positions 3 and 6, allowing the identification of viruses in the mix. The identity of viruses found in upper noninoculated leaves of each infected plant is shown below each chromatogram.

The attenuation produced by increasing UpA frequencies suggests that, conversely, variants fitter than the original PPV isolate may be generated if frequencies are reduced below WT levels. As shown above, we were, however, unable to detect statistically significant differences among F3_UpA_low, F4_UpA_low, and SD-PPV in single infection experiments (Fig. 1 and Fig. S2). The accumulation of a combined UpA low mutant containing a 2-kb segment of F3 and F4 (F3-4_UpA_low, 24 UpA sites) was similarly comparable to that of SD-PPV (88 UpAs in this region) (data not shown). Given that subtle differences in viral fitness might not be perceived in single infection comparisons, we carried out a competition assay, which is a more stringent test based on the coinfection of two competing viruses in a single plant (Fig. 3A). Hence, we compared the accumulations of the F3-4_UpA_low mutant and the SD-PPV WT in N. benthamiana plants inoculated by biolistics, since this method allowed precise calculation of the proportion of each infectious cDNA clone in the inoculum. Two independent mixes (mix 1 and mix 2) as biological replicates were prepared with twice the amount of parental pLX-SD-PPV, to provide a more stringent test of fitness enhancement. Analysis of chromatogram peaks of sequenced PCR products from inocula confirmed that the amount of pLX-SD-PPV in these mixes was higher than that of pLX-F3-4_UpA_low (Fig. 3B). The analysis of reverse transcription-PCR (RT-PCR) products from upper noninoculated leaves of the two plants infected with mix 1 revealed the presence of both competing viruses (Fig. 3B). However, the equivalent analysis in parallel of plants infected with mix 2 indicated that F3-4_UpA_low fully outcompeted SD-PPV (Fig. 3B). Extracts from plants infected with mix 1 were used to inoculate two healthy plants, and RT-PCR-amplified products from systemically infected leaves of three plants of this passage were sequenced. F3-4_UpA_low was the only virus detected in these tissues (Fig. 3B and Fig. S3). Combined, these results demonstrate that a plant virus with lower UpA frequency is fitter *in vivo* than its parental control.

10.1128/mBio.02818-19.3 FIG S3 Competition between a low-UpA virus and its parental control after one passage. Images show a representative region of the chromatogram obtained from the sequencing of PCR products from viral progeny cDNAs. This region includes two SNPs in positions 3 and 6, allowing the identification of viruses in the mix. The identity of viruses found in upper noninoculated leaves of each infected plant is shown below each chromatogram. Download FIG S3, JPG file, 0.1 MB. Copyright © 2020 González de Prádena et al. 2020 González de Prádena et al. This content is distributed under the terms of the Creative Commons Attribution 4.0 International license.

### UpA-rich viruses are genetically stable.

**FIG 4:**
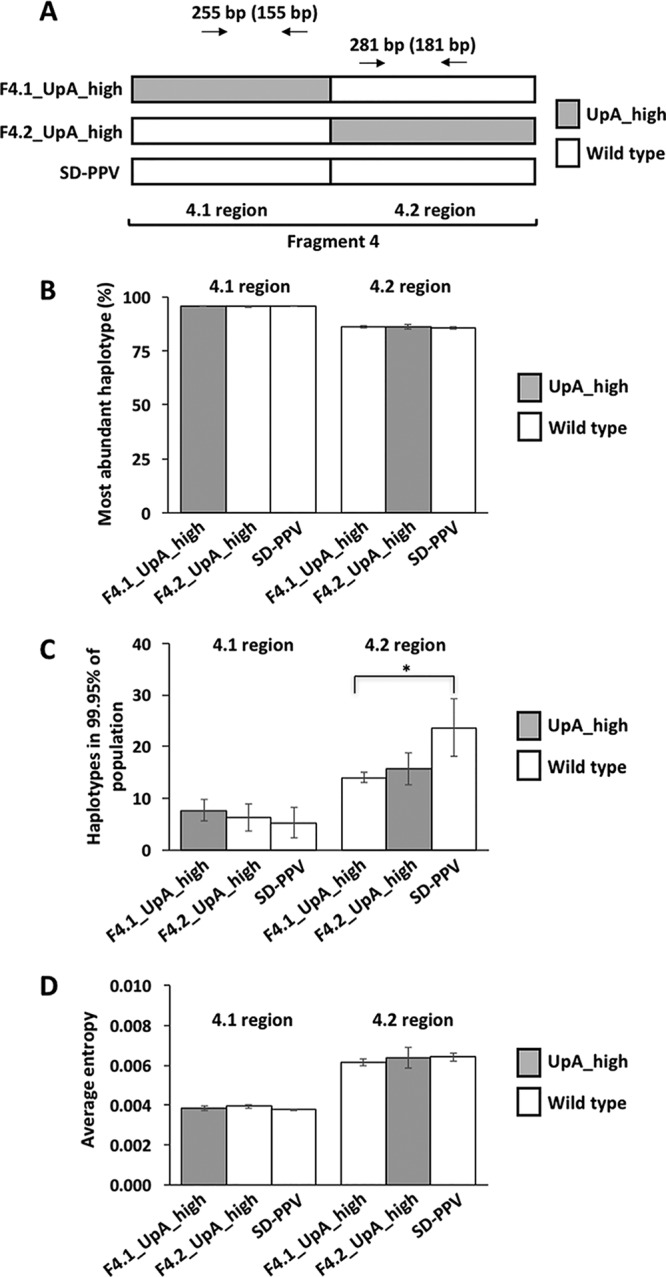
Genome stability of attenuated SD-PPV with high UpA content. (A) Schematic representation of the viral segment F4 (divided into F4.1 and F4.2) from the parental SD-PPV and those viral derivatives used for this experiment. Virus genotypes in F4 (based on UpA content) are indicated with white or gray boxes. The two regions from which RT-PCR products were obtained, and the size of these amplicons, are also indicated. The sizes of amplicons after quality trimming are indicated in parentheses. (B) Bar plots representing the percentage of the most abundant viral haplotype in upper noninoculated leaves of plants infected with the indicated viruses. (C) Bar plots representing the numbers of different viral haplotypes in 99.95% of the viral population in upper noninoculated leaves of plants infected with the indicated viruses. Asterisks indicate significant differences (*P* value < 0.05, *t* test). (D) Bar plots representing the overall entropy of the region under study when considering every position after the trimming of sequenced amplicons (155 for 4.1 region and 181 for 4.2 region). All bars represent the mean ± SD of results for three infected plants (*n* = 3), and they are depicted in white or gray based on the genotype of the corresponding viruses in the indicated regions.

It has been proposed that synthetic attenuated virus engineering by genome-scale recodification is ideal for designing live vaccines in mammals, as the recovery of virulence is quite unlikely since it relies on dozens or potentially hundreds of reversions (10). To investigate whether attenuated SD-PPV variants with high UpA frequency were genetically stable in both mutated and wild-type genome sequences, we used the replication-proficient F4.1_UpA_high and F4.2_UpA_high mutants, which showed intermediate degrees of attenuation (Fig. 2C), and the parental SD-PPV as the control (Fig. 4A). Upper noninoculated leaves of three independent N. benthamiana plants per treatment were harvested at 21 dpi, and amplicons were generated by RT-PCR (Fig. 4A). After MiSeq sequencing of the so-produced DNA fragments, we explored the haplotypes in each viral progeny to assess variability. We found that (i) regardless of the virus analyzed and the sequenced region, the most abundant haplotype, by far, had the same sequence as the inoculated viral cDNA; (ii) the frequencies of this particular molecular species were almost identical in the two mutants and the control virus in both sequenced regions (Fig. 4B); and (iii) the numbers of different viral haplotypes contributing to 99.95% of the quasispecies were comparable among different viruses in the analyzed regions. In fact, the parental SD-PPV displayed a significantly larger number of haplotypes in one of the fragments (*P* value < 0.05, *t* test) than the F4.1_UpA_high variant (Fig. 4C). For another estimate of genome stability, we also calculated the overall entropy of sequenced fragments, and in both modified and unmodified segments, values were very low and similar for all viruses (Fig. 4D). The combined data demonstrated that UpA-rich SD-PPV variants (i) do not evolve toward viral species with lower UpA frequency and (ii) display an overall variability equivalent to that of the parental control.

### RNA silencing-independent restriction of UpA-rich PPV.

**FIG 5:**
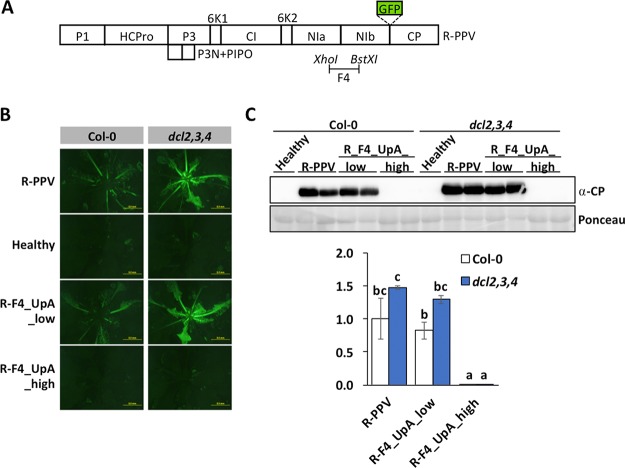
Attenuation of a UpA-rich virus is not alleviated in the *A. thaliana*, RNA silencing knockout, *dcl2*,*3*,*4* mutant. (A) Schematic representation of R-PPV (each box represents a viral cistron). The genome segment F4 is indicated. (B) Images taken with an epifluorescence microscope of *A. thaliana* Col-0 plants (wild type and *dcl2*,*3*,*4* mutant) infected with the indicated viruses at 15 days postinoculation (dpi). Bars, 5 mm. (C) PPV CP-specific immunoblot analysis of extracts from upper noninoculated leaves collected at 15 dpi from *A. thaliana* plants agroinoculated with the indicated PPV variants. The membrane stained with Ponceau red that shows the RubisCO large subunit was included as a loading control. A bar graph showing the mean ± SD (*n* = 2) of immunoblot signals in arbitrary units is given below the immunoblot (for comparison, we considered the average for R-PPV to be 1).

In plants, the RNA silencing mechanism works, among many other tasks, as the main defensive barrier against RNA viruses (19). In order to know whether this pathway is directly or indirectly implicated in the attenuation of UpA-rich viruses, we analyzed the accumulation of modified viral variants in the Arabidopsis thaliana *dcl2*,*3*,*4* triple mutant, which lacks an active antiviral RNA silencing system (20, 21). For this particular experiment, we used R-PPV as the wild-type control and parental virus to harbor the modified F4 fragments, as this PPV strain is well adapted to herbaceous hosts and, consequently, more sensitive to any potential improvement in viral fitness in *A. thaliana*. Hence, the pLX-R-PPV infectious clone and derivative mutants having either low or high UpA frequencies in segment F4 (R-F4_UpA_low and R-F4_UpA_high, respectively) (Fig. 5A) were agroinoculated in wild-type and *dcl2*,*3*,*4 A. thaliana* Col-0 plants. Observations under UV light revealed high accumulations of GFP at 15 dpi in upper noninoculated leaves of those plants inoculated with R-PPV and R-F4_UpA_low, with consistently stronger fluorescent signals in the case of the silencing-defective *dcl2*,*3*,*4* plants (Fig. 5B). In contrast, plants inoculated with the R-F4_UpA_high mutant, irrespective of their genotype, displayed no fluorescence at all (Fig. 5B). The estimation of virus accumulation in upper noninoculated leaves by PPV CP immunoblotting (Fig. 5C) agreed with GFP observations, indicating that R-F4_UpA_high is unable to infect not only wild-type *A. thaliana* plants but also knockout mutants lacking the RNA silencing-mediated antiviral pathway.

### UpA enrichment reduces the accumulation of an RNA produced by the host RNA polymerase II.

**FIG 6:**
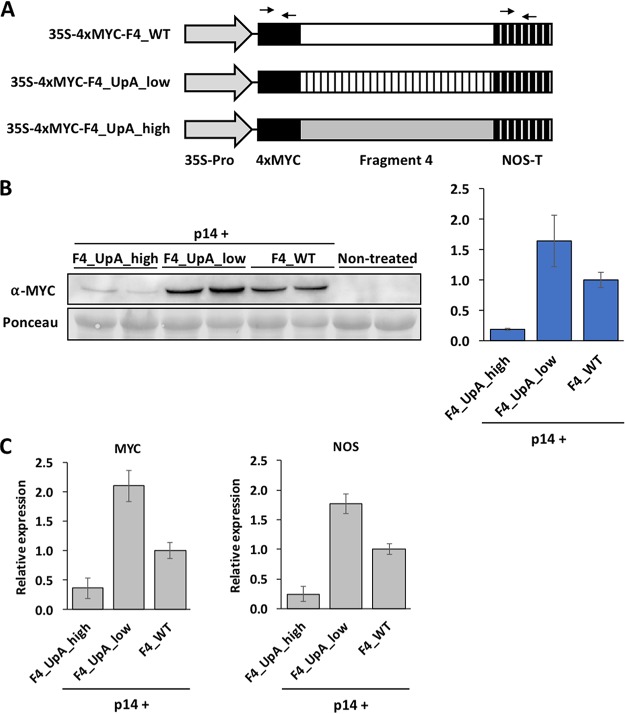
Reduced accumulation of a UpA-rich RNA transcribed by the host RNA polymerase II. (A) Schematic representation of constructs transiently expressed by coagroinfiltration along with the P14 silencing suppressor. Different parts of the constructs, including the 4×MYC tag, are indicated, as well as those targeted regions in 4×MYC and NOS terminator (NOS-T) amplified for RT-qPCR analyses. (B) MYC-specific immunoblot analysis of extracts from agroinfiltrated leaf patches of N. benthamiana plants at 7 days postagroinfiltration (dpa). The membrane stained with Ponceau red that shows the RubisCO large subunit was included as a loading control. A bar graph showing the mean ± SD (*n* = 2) of immunoblot signals in arbitrary units is to the right (for comparison, we considered the average for F4_WT to be 1). (C) Accumulation of RNAs, estimated by RT-qPCR, corresponding to the indicated F4 fragments from agroinfiltrated leaf patches of N. benthamiana plants at 7 dpa. The result obtained for two independent pairs of primers (MYC and NOS) is depicted. Bar graphs show the mean ± SD (*n* = 2 plants) relative expression (for comparison, we considered the average for F4_WT to be 1).

We also wondered whether the suppression of UpA-rich RNAs is limited to virus-derived molecules or also affects other RNA species. To address this question, F4 segments encoding the same protein with different UpA frequencies (wild type and low- and high-UpA mutants) (Fig. 6A) were transiently expressed in N. benthamiana leaves by agroinfiltration. In this case, transcription is under the control of the strong 35S promoter, which drives the production of RNAs by RNA polymerase II in the nucleus of infiltrated plant cells. To allow their detection by immunoblotting, the F4-derived proteins were tagged with a 4×MYC epitope at their N termini. These proteins were coexpressed with pothos latent virus (PoLV) P14, a potent RNA silencing suppressor, aiming to avoid the well-known effect of the classical RNA silencing over all transgenes. The estimation of protein accumulation by immunoblotting against the 4×MYC tag at 7 dpi showed a much lower expression of 4×MYC-F4 when the protein was expressed from the UpA-rich RNA (Fig. 6B). We then tested by RT-quantitative PCR (RT-qPCR) whether this result is due to differences in RNA accumulation. Interestingly, and irrespectively of the RNA region targeted for the qPCR analysis (either MYC or NOS-T), we found that the yield of the UpA-high RNA is much lower than that of the other analyzed species (Fig. 6B). As these differences cannot be explained by the amount of DNA delivered into plant cells by agroinfiltration in each treatment (Fig. S4), this result indicates that the yield of the UpA-high RNA is selectively restricted when it is produced by the host RNA polymerase II. The presence of PoLV P14 warrants that consistently with our previous finding with modified viruses (Fig. 5), the lower accumulation of the UpA-high 4×MYC-F4 RNA is independent of the conventional RNA silencing pathway. Remarkably, in line with the enhanced fitness of the PPV variant with lower UpA content (Fig. 3), the 4×MYC-F4 RNA with lower UpA frequency accumulated in significantly larger amounts than its wild-type counterpart (Fig. 6B).

10.1128/mBio.02818-19.4 FIG S4 qPCR to estimate the amount of DNA introduced in N. benthamiana leaves by agroinfiltration. Tested samples correspond to those analyzed in Fig. 6. The result obtained for two independent pairs of primers (MYC and NOS) is depicted. Bar graphs show the mean ± SD (*n* = 2 plants). For comparison, we considered the average for F4_WT to be 1. Download FIG S4, JPG file, 0.05 MB. Copyright © 2020 González de Prádena et al. 2020 González de Prádena et al. This content is distributed under the terms of the Creative Commons Attribution 4.0 International license.

## DISCUSSION

CpG/TpA constraints were reported a long time ago in animal and plant genomes (22–26), but reasons for this phenomenon remain obscure. Recently, however, the molecular underpinning of CpG restriction in animal RNAs was discovered: a protein termed ZAP recognizes, and triggers the depletion of, RNAs with high CpG content (13). Remarkably, a further involvement of ZAP in the recognition of UpA-rich RNAs has been described more recently (14). This particular study has even shown that not only ZAP, but also a functional OAS3/RNAseL system, is required for restricting the replication of animal viruses with high frequencies of CpG and UpA dinucleotides (14). Consequently, the observed CpG and UpA constraints in the genome of animal RNA viruses may serve to mimic dinucleotide frequencies of host RNAs in order to escape from the action of ZAP and OAS3-coupled restriction pathways.

The work that we present here in plants definitively demonstrates that the frequency of UpA profoundly influences the accumulation of plant RNA viruses, whereas that of CpG has a marginal effect, if any. On the one hand, we found that the attenuation due to high UpA frequency takes place in two different PPV backgrounds (SD-PPV and R-PPV) in three tested hosts (N. benthamiana, *P. persica*, and *A. thaliana*), suggesting that it could be a general feature of any plant-virus combination. Among the results with UpA-modified PPV variants, those of the competition experiment are the most intriguing. Replication enhancement was indeed previously observed by reduction of UpA frequency in echovirus 7 (*Picornaviridae* family) in human cells (11, 12). Why viruses have not naturally evolved a lower UpA composition to increase its accumulation is not clear. As previously suggested in the case of echovirus 7 (12), since the overall viral fitness is the result of a multitude of factors, the frequency of UpA might be fine-tuned to maximize other aspects of viral fitness not captured in the infection models used in the current study.

On the other hand, the attenuation of CpG-rich PPV was much less pronounced in N. benthamiana and was undetected in *P. persica*, where the CpG-high virus displays fitness similar to that of the parental control, suggesting that the restriction of CpG-high viruses may be host specific. All in all, the absence of known functional homologues of ZAP and the OAS3/RNAseL system in plants, the lower restriction of CpG in the genome of potyvirids, and the weak (or non-) attenuation of F3_CpG_high and F4_CpG_high PPV variants shown in the current study suggest that cellular pathways underlying dinucleotide restriction in plants may differ from those operating in animals.

Our efforts to gain insight about how UpA-rich viruses are restricted in plants led us to find that this attenuation is not related to the classical RNA silencing pathway, the main system against viruses in plants. Given the good correlation between the fitness of manipulated PPV variants and the accumulation of independent F4 RNA fragments, we hypothesize that the same pathway restricts the accumulation of both virus- and RNA polymerase II-derived UpA-rich RNAs. Since these two types of RNAs (PPV RNAs and RNA polymerase II transcripts) overlap only spatially in the cytoplasm, we also speculate that the proposed restriction operates in this compartment. In fact, the idea that a specific RNase hydrolyzes UpA-rich RNAs in the cytoplasm has been postulated and studied *in vitro* with a macrophage cytoplasmic RNase as long ago as 1989 by Beutler and collaborators, as they noticed that the presence of TpA is particularly constrained in DNA destined to produce RNA that will accumulate in the cytosol (e.g., mRNAs, rRNAs, tRNAs) (27). Hence, our finding is in perfect agreement with this observation, and it might constitute the first evidence of such an alternative RNA silencing pathway governed by the UpA dinucleotide frequency in RNAs that are produced/located in the cytoplasm. Like the low content of UpA and CpG in animal viruses (see above), the low frequency of UpA observed in the genome of plant RNA viruses might then constitute the response of these pathogens to escape from a plant antiviral system that identifies self from nonself RNAs based on the dinucleotide content. Therefore, our future work will be focused first on determining the step(s) of the infection cycle targeted by the UpA content-related restriction. Then, the identification of plant factors involved in UpA recognition and subsequent RNA processing will be, indeed, a main goal in order to fully understand the role of the proposed host pathway and the way that it works.

Irrespective of mechanisms, however, the demonstrated impact of dinucleotide frequency modification in the genomes of plant viruses (and the great stability of this effect over the time) opens the possibility of developing systematic approaches based on dinucleotide manipulation to control viral fitness for different purposes. Cross-protection (the equivalent of vaccination in mammals), virus-induced gene silencing, and potential enhancement of heterologous expression through compositionally modified virus mutants, vectors, and transgenes are all areas that may be exploited in the future.

## MATERIALS AND METHODS

### Calculation of OR.

Full-length sequences of the indicated viruses were downloaded from NCBI (virus names and genome references are indicated in Tables S1, S2, S3 and S4 in the supplemental material). OR of each dinucleotide (*xy*) was calculated with the formula OR*_xy_* = *f_xy_*/(*f_x_* × *f_y_*) (where *f* is frequency) by using the program Composition Scan in the SSE package v1.3 (28). The cutoff values to estimate underrepresentation and overrepresentation of the OR were established as 0.78 and 1.23, respectively (16).

### RNA structure prediction and sequence variability.

A total of 156 coding region complete sequences of PPV were downloaded from GenBank. Sequences were filtered to 1% nucleotide divergence to leave 61 sequences available under the following accession numbers: Y09851, HQ840518, KY221840, KJ787006, KC020124, KC347608, HG916856, HG916858, HG916860, LN852400, JN596110, HG916861, HG916859, HG916862, HQ670746, AY912055, AM157175, DQ431465, AY028309, KM273015, JX013532, EU117116, GU474956, EF640935, X16415, KP998124, X81083, D13751, LT600780, KR028387, LC331298, LC333268, LC333552, HF585098, KR028385, KR006730, KR028386, LT158756, MF371000, EU734794, LC228949, M92280, FM955843, HF585099, AJ243957, HF585101, MF370990, MF370991, MF370985, MF370986, MF370994, MF370987, MF370992, MF371002, MF370993, MF370989, MF371003, MF370996, MF370997, MF370984, MF370995. Aligned sequences were scanned for RNA secondary structure with the program Folding Energy Scan in the SSE package, using 270 base fragments increasing by 15-base increments and 50 sequence-order-randomized controls (NDR algorithm). Mean MFED (minimum folding energy differences) values for each fragment were plotted against the midpoint of each fragment to localize areas of sequence-order-dependent RNA secondary structure. Synonymous sequence variability was determined by measurement of mean pairwise distances between successive fragments of 300 bases, with 30-base increments between fragments, using the program Sequence Scan in the SSE package.

### Design of modified PPV genome segments.

A 5-kb central region of the PPV genome was divided into five segments (F1, F2, F3, F4, and F5) of around 1 kb each (Fig. 1B). For permuted mutants, we designed five synthetic DNA fragments (one per segment) with the algorithm CDLR in Sequence Scramble in the SSE package and by following these criteria: randomized nucleotides, but maintaining protein coding and frequencies of each dinucleotide identical to the native PPV sequence. For CpG/UpA mutants, we designed eight synthetic DNAs corresponding to fragments F3 and F4 (four per segment) in order to (i) boost CpG (CpG_high), (ii) suppress CpG (CpG_low), (iii) boost UpA (UpA_high), and (iv) suppress UpA (UpA_low) without changing the encoded amino acids. Additional DNAs corresponding to F4 were also synthetized as controls, and they were designed with the following criteria: (i) boost UpA while the frequency of U and A mononucleotides is kept as in wild-type F4 (UpA_high_M_fixed); (ii) boost U and A mononucleotides as in F4_UpA_high while the frequency of UpA is kept as in wild-type F4 (F4_U+A_high_UpA_fixed); and (iii) boost ApUs until they reach the same frequency as UpAs in F4_UpA_high_M_fixed while the frequency of UpA is kept as in wild-type F4 (F4_ApU_high_UpA_fixed). None of these modifications alter the amino acids encoded in F4. Dinucleotide mutants were designed with the program Sequence Mutated in the SSE package. Values for relevant features of all synthetic PPV segments were calculated with the SEE package, with the exception of the codon adaptation index (CAI) for N. benthamiana codon usage, which was evaluated by using the tool available at the website http://genomes.urv.es/CAIcal/ (29). All of these values are shown in Table S5.

### Infectious cDNA clones.

Modifications were introduced in the infectious cDNA clones pLX-PPV (30), here named pLX-R-PPV, and pLX-SD-PPV, which produce two GFP-tagged variants of PPV. Particularly, SD-PPV is a chimera from PPV-R and PPV-D strains, which, unlike these two PPV strains, is able to properly infect N. benthamiana, a model plant, and *P. persica*, a natural host of PPV (31). The fitness of SD-PPV is lower than that of (i) PPV-R in N. benthamiana and (ii) PPV-D in *Prunus persica*, so that little differences in virus accumulation due to the introduced modifications should be observed and quantified more easily in both hosts. The infectious cDNA clone pLX-SD-PPV was built by replacing the ScaI/SalI 4,767-bp fragment from pLX-R-PPV with the equivalent fragment from pICPPV-5′BD (31).

Reengineered viral cDNA segments were synthesized (GeneArt; Life Technologies) and then excised from the provided intermediate plasmids with the same restriction enzymes that were later used for exchanging the equivalent wild-type segments in pLX-SD-PPV (Fig. 1B) and pLX-R-PPV (Fig. 5A). There is no unique restriction site between F1 and F2; therefore, we used overlapping PCRs to generate two hybrid F1+F2 products: (i) F1permuted-F2wt and (ii) F1wt-F2permuted. PCRs were carried out with primer pairs 3340/3364 (to amplify F1, with either wild-type or permuted F1 as the template), 3365/3341 (to amplify F2, with either wild-type or permuted F2 as the template), and, finally, with pair 3340/3341 (to amplify F1+F2, with the adequate mix of overlapping PCRs as the template). The two produced F1+F2 segments were digested with SalI and SfoI to be further exchanged with the equivalent wild-type fragment in pLX-SD-PPV (Fig. 1B). The same strategy was used to divide segment F4 in F4_UpA_high in order to produce F4.1_UpA_high and F4.2_UpA_high. In this case, PCRs were carried out with primer pairs 3344/3392 (to amplify F4.1, with either the wild type or F4 with high UpA as the template), 3393/3345 (to amplify F4.2, with either the wild type or F4 with high UpA as the template) and, finally, with pair 3344/3345 (to amplify F4.1+F4.2, with the adequate mix of overlapping PCRs as the template). The two produced F4.1+F4.2 segments were digested with XhoI and BstXI to be further exchanged with the equivalent wild-type fragment in pLX-SD-PPV (Fig. 1B).

The sequences of all primers used for this study are listed in Table S6.

10.1128/mBio.02818-19.10 TABLE S6 List of primers used in this study. Download Table S6, PDF file, 0.05 MB. Copyright © 2020 González de Prádena et al. 2020 González de Prádena et al. This content is distributed under the terms of the Creative Commons Attribution 4.0 International license.

### Binary plasmids for transient expression.

Wild-type, UpA_low, and UpA_high versions of the F4 segment were amplified by PCR with the primer pair 3513/3514, which adds a stop codon. These products were cloned with Gateway technology in the destination vector pGWB718 (35S promoter, 4×MYC tag at the N terminus of the gene of interest, NOS terminator) (32), using pDONR207 as the intermediate, by following the manufacturer’s instructions (Invitrogen).

### Plant infection, fluorescence imaging, and transient expression.

Plants were grown in a greenhouse with 16-h-light/8-h-dark cycles at 20 to 24°C for N. benthamiana and *P. persica* GF305 and 8-h-light/16-h-dark cycles at 21°C for *A. thaliana*. Leaves of 4-week-old plants were infiltrated using Agrobacterium tumefaciens C58C1 strains carrying the indicated plasmids, as previously described (33). Just before agroinoculation, cultures were adjusted to an optical density at 600 nm (OD_600_) of 0.05 for infiltrations in N. benthamiana, an OD_600_ of 0.5 for infiltrations in *P. persica*, and an OD_600_ of 1.0 for infiltrations in *A. thaliana*. When required, N. benthamiana plants were inoculated by bombardment with microgold particles coated with DNA of the indicated plasmids by using a Helios gene gun system (Bio-Rad), as previously described (34). For viral passages, young N. benthamiana plants were dusted with carborundum and then finger-rubbed with 15 μl of crude extract from the indicated systemically infected plant tissues (1 g in 2 ml of 5 mM sodium phosphate, pH 7.2). Virus-derived GFP fluorescence was observed in a Leica MZ FLIII stereomicroscope (Leica Microsystems), and images were acquired as described previously (35). For transient expression, two leaves from 1-month-old N. benthamiana plants were infiltrated with A. tumefaciens as previously described (36). For this particular work, a 1:1 mix of A. tumefaciens strains carrying the indicated pGWB718-derived plasmid and pBin61-P14, with both cultures at an OD_600_ of 0.5, were infiltrated.

### Competition assay.

DNA mixtures of the indicated infectious clones were inoculated by biolistics as described above. The identity of viruses in upper noninoculated leaves was determined by RT-PCR followed by Sanger sequencing. In brief, total RNA was first isolated from systemically infected tissues with a FavorPrep plant total RNA purification minikit (Favorgen Biotech), by following the manufacturer’s instructions. Second, around 500 ng of total RNA was subjected to reverse transcription with the Moloney murine leukemia virus (M-MuLV) enzyme (New England BioLabs) and random hexanucleotides as primers, and the so-generated cDNAs were then used as the template to amplify the whole F4 segment (primer pair 3463/3345). Finally, PCR products were Sanger sequenced (Macrogen Europe).

### High-throughput sequencing and data processing.

Two amplicons were generated by RT-PCR, as explained above, from upper noninoculated leaves of N. benthamiana plants infected with the indicated viruses and harvested at 21 dpi. The first amplicon spans a region of segment F4.1 (255 nucleotides [nt], primers 3464/3465), whereas the second one spans a region of segment F4.2 (281 nt, primers 3466/3467). These PCR products were then subjected to two additional rounds of PCR, with a low number of cycles, to attach the appropriate adaptors and barcodes at their 5′ and 3′ ends. Paired-end sequencing (2 × 300) was done with a MiSeq reagent kit v3 on a MiSeq sequencing platform (Illumina) by following the manufacturer’s instructions.

To reduce the number of reads with sequencing errors, we first used Trimmomatic (37) to trim the first and last 50 nt from the raw data and to remove low-quality pairs. Second, we used FLASH (38) to keep only those forward reads (R1) showing perfect reverse complementarity with their corresponding reverse reads (R2). The number and frequency of different filtered R1 species were calculated with an in-house script. These reads were further aligned against the sequence of their corresponding infectious cDNA clones with BBMap (B. Bushnell, https://sourceforge.net/projects/bbmap/). These alignments were analyzed with SAMtools (39) to then create a list with the nucleotide frequency per sequenced position by using an in-house script. We then calculated Shannon entropy per each position [*H* = −∑*i* (p*_i_* × ln p*_i_*); where *H* is the Shannon entropy at a given position, and p*_i_* is the probability of having any of the four nucleotide (*i*) at that position], and used the average of *H* to estimate variability per amplicon (40).

In-house scripts are available upon request.

### Immunoblotting.

Crude extracts were obtained from N. benthamiana, *P. persica*, and *A. thaliana* by homogenization of ground frozen leaf tissues in disruption buffer (50 mM Tris-HCl, pH 7.5, 6 M urea, 2% SDS, and 5% β-mercaptoethanol). After centrifugation at 13,000 × *g* for 10 min, samples were boiled for 5 min at 95°C. Proteins were separated in SDS-PAGE gels (12% acrylamide) and electroblotted onto a nitrocellulose membrane. The CP from PPV was detected with a specific rabbit serum used as the primary antibody and horseradish peroxidase (HRP)-conjugated goat anti-rabbit IgG (Jackson ImmunoResearch) as the secondary reagent. MYC-tagged proteins were detected with a specific anti-MYC monoclonal antibody (AbMART) and HRP-conjugated sheep anti-mouse IgG (Sigma) as the secondary antibody. Immunostained proteins were visualized by enhanced chemiluminescence detection with Clarity ECL Western blotting substrate (Bio-Rad) in a ChemiDoc system (Bio-Rad). Band intensity in arbitrary units was estimated by using Image Lab software (v.6.0.0), with the signal of one selected sample considered as the reference. Ponceau red staining of membranes was used to check the global protein content of samples.

### Reverse transcription followed by quantitative PCR.

Total RNA was isolated from the indicated ground leaf tissue, and cDNA was synthesized as described above. The abundance of specific transcripts was measured by probing the cDNA by quantitative PCR using EvaGreen master mix (Solis Biodyne), in a 7500 real-time PCR system (Applied Biosystems), with primers 3519/3520 (MYC) or 3527/3528 (NOS). The transcript levels between samples were normalized against the expression of PP2A housekeeping gene (41) using primers 2806/2807.

### Data availability.

MiSeq raw data are available in ArrayExpress under accession number E-MTAB-8399.
