# Supplementary material for: Plant Virus Genome Is Shaped by Specific Dinucleotide Restrictions That Influence Viral Infection
Source: mBio. 2020 Feb 18;11(1):e02818-19. doi: 10.1128/mBio.02818-19 (PMC7029135; doi:10.1128/mBio.02818-19)
Supplement: TABLE S2 [file mBio.02818-19-st002.pdf]

Table S2. Odds ratio for members of the family *Alphaflexiviridae*

Genus	ID	Abrev.	Name	AA	AT	AC	AG	TA	TT	TC	TG	CA	CT	CC	CG	GA	GT	GC	GG	Size	A	T	C	G
Allexvirus	KY696659.1	AVS	Alfalfa virus S	1,0202	0,844	1,0522	1,0537	0,7309	1,233	0,9052	1,2706	1,1256	1,1208	0,962	0,7618	1,0597	0,8113	1,0772	0,9968	8349	0,29	0,21	0,30	0,21
	KX058345.1	APV	Arachis pintoi virus	1,0198	0,8345	1,0098	1,1077	0,6567	1,235	0,9659	1,2819	1,1689	1,0878	0,9979	0,7053	1,061	0,8932	1,021	0,9893	7599	0,29	0,20	0,29	0,22
	JN053266.1	BVE	Blackberry virus E	0,9458	0,8481	1,0703	1,104	0,7096	1,1635	1,0027	1,2009	1,2083	1,1067	0,9136	0,7741	1,0337	0,886	1,0353	1,0102	7718	0,27	0,20	0,31	0,22
	AB010300.1	GVA	Garlic virus A	1,0451	0,8074	1,0939	1,0176	0,7676	1,1205	0,9763	1,2289	1,014	1,2104	0,889	0,9044	1,18	0,8295	1,0552	0,8547	8657	0,28	0,23	0,29	0,20
	KM379144.1	GVB	Garlic virus B	0,9906	0,9076	1,1078	0,9804	0,7187	1,1706	0,9786	1,2443	1,1582	1,0992	0,8885	0,805	1,1212	0,8237	1,0085	1,0057	8336	0,30	0,23	0,26	0,21
	AB010302.1	GVC	Garlic virus C	1,0047	0,8613	1,1402	0,9667	0,6915	1,2323	0,9078	1,3009	1,1794	1,0661	0,8799	0,8262	1,0996	0,8606	1,0535	0,9429	8405	0,30	0,23	0,27	0,21
	KF555653.1	GVD	Garlic virus D	1,0024	0,8114	1,1478	1,0098	0,7615	1,1782	0,866	1,3169	1,088	1,1594	0,9479	0,7736	1,1358	0,883	0,9964	0,9356	8424	0,30	0,22	0,27	0,21
	AJ292230.1	GVE	Garlic virus E	0,9743	0,8757	1,1218	1,0011	0,7426	1,175	0,9135	1,2711	1,0876	1,099	0,9632	0,8358	1,1739	0,8656	0,9741	0,9398	8451	0,29	0,22	0,28	0,22
	U89243.1	GVX	Garlic virus X	0,9836	0,9336	1,092	0,9841	0,7167	1,241	0,9108	1,2612	1,1848	1,0129	0,8944	0,8444	1,1042	0,8228	1,0909	0,9294	8106	0,31	0,23	0,26	0,21
	M97264.1	SVX	Shallot virus X	1,0351	0,881	1,0542	1,0028	0,7098	1,1553	1,0118	1,2199	1,1138	1,1334	0,8998	0,838	1,1064	0,8141	1,0514	0,9817	8831	0,29	0,22	0,29	0,21
	MF150239.1	VLV	Vanilla latent virus	0,9747	0,9235	1,0437	1,0765	0,7257	1,17	1,014	1,2687	1,2189	1,0695	0,9083	0,6507	1,0678	0,836	1,0344	1,0277	7462	0,33	0,22	0,26	0,19
Lolavirus	EU489641.1	LLV	Lolium latent virus	0,9227	0,9376	1,019	1,1241	0,7599	1,1158	0,9631	1,2365	1,1977	1,131	1,0085	0,6356	1,0531	0,8102	0,999	1,1024	7674	0,28	0,20	0,29	0,23
Mandarivirus	KP313240.1	CYVCV	Citrus yellow vein clearing virus	1,0938	0,822	1,0783	0,9226	0,7551	1,1231	0,9634	1,2876	1,0077	1,2415	0,9463	0,8224	1,1116	0,7262	1,0152	1,1003	7531	0,28	0,21	0,32	0,19
	AF406744.1	ICRV	Indian citrus ringspot virus	1,0439	0,8812	1,0639	0,9544	0,7139	1,0926	1,0598	1,215	1,0786	1,1556	0,9177	0,8652	1,1014	0,819	0,984	1,0643	7560	0,28	0,20	0,32	0,20
Platypuvirus	KC923234.1	DOSV	Donkey orchid symptomless virus	1,0534	1,036	0,9856	0,9298	0,6029	1,0458	1,1838	1,1341	1,0981	1,0436	0,883	1,0212	1,1577	0,8569	1,0177	0,9271	7838	0,25	0,20	0,32	0,23
Potexvirus	KR872420.1	ActVX	Actinidia virus X	1,0088	0,8278	1,0368	1,1119	0,7014	1,0952	0,9638	1,3299	1,1623	1,1687	0,9583	0,6833	1,0644	0,8877	1,0488	0,9645	6888	0,27	0,22	0,30	0,21
	FJ670570.2	AllVX	Allium virus X	1,018	0,8356	1,0602	1,0546	0,6243	1,1927	1,0908	1,1786	1,1443	1,1754	0,8887	0,787	1,1511	0,7619	0,9905	1,0559	7118	0,28	0,21	0,31	0,20
	AY863024.1	AMV	Alternanthera mosaic virus	1,0619	0,9087	0,8516	1,2054	0,5478	1,2255	1,175	1,0986	1,2048	1,0885	1,0481	0,6046	1,1077	0,7764	0,9457	1,1598	6607	0,27	0,22	0,29	0,22
	AB206396.1	AlsVX	Alstroemeria virus X	1,1078	0,8423	1,0059	0,9838	0,6146	1,1646	1,0954	1,2599	1,1227	1,063	0,9466	0,8407	1,0164	0,9727	0,9854	1,0271	7009	0,29	0,19	0,32	0,19
	NC_010416.1	AV3	Asparagus virus 3	1,1149	0,9127	0,9894	0,9745	0,6027	1,1006	1,128	1,1425	1,0833	1,0741	0,9434	0,9242	1,1326	0,877	0,9751	1,0108	6935	0,24	0,21	0,35	0,21
	D26017.1	BMV	Bamboo mosaic virus	1,0584	0,8771	1,0411	0,9683	0,7522	1,1717	0,9822	1,2258	1,0867	1,1931	0,9669	0,7471	1,0202	0,7496	1,0045	1,1932	6366	0,30	0,19	0,30	0,21
	AF308158.2	CVX	Cactus virus X	1,0617	0,8787	0,947	1,121	0,5627	1,1067	1,1685	1,2091	1,1833	1,2126	0,9774	0,5806	1,1105	0,7459	0,9279	1,2216	6614	0,27	0,22	0,30	0,21
	U23414.1	CCMV	Cassava common mosaic virus	1,0608	0,8746	0,9553	1,098	0,5823	1,1384	1,1289	1,2912	1,2348	1,3016	0,9098	0,4632	1,0693	0,6765	1,0344	1,1974	6376	0,31	0,22	0,26	0,22
	KY288487.1	CVX	Cassava virus X	1,0553	0,95	0,9358	1,0611	0,7375	1,0876	0,9711	1,2837	1,1284	1,1685	1,0597	0,5662	1,064	0,759	1,0445	1,1348	5879	0,28	0,24	0,26	0,21
	D29630.1	CYMV	Clover yellow mosaic virus	0,9971	0,879	0,9279	1,2342	0,7405	1,0817	1,1361	1,1329	1,0816	1,1943	1,0841	0,5484	1,1302	0,8193	0,8569	1,1835	7015	0,32	0,19	0,30	0,19
	U62963.1	CMV	Cymbidium mosaic virus	1,131	0,9809	0,9532	0,9173	0,8429	1,011	0,9197	1,314	0,9822	1,1423	1,0398	0,7895	1,0457	0,803	1,1047	1,0291	6227	0,27	0,25	0,29	0,20
	M62730.1	FMV	Foxtail mosaic virus	1,0535	0,8321	1,1284	0,9202	0,671	1,1973	1,015	1,1928	1,1103	1,1462	0,8653	0,9134	1,0889	0,8325	1,0098	1,0325	6151	0,27	0,21	0,30	0,23
	AJ620114.1	HVX	Hosta virus X	1,0896	0,7927	0,9681	1,0937	0,5455	1,2786	1,2326	1,0569	1,1534	1,2215	0,9262	0,7165	1,0517	0,748	0,9499	1,2066	6528	0,29	0,18	0,30	0,22
	AY707100.1	HRV	Hydrangea ringspot virus	1,0933	0,778	0,9168	1,204	0,5637	1,1992	1,2165	0,969	1,0401	1,2216	1,0387	0,725	1,1805	0,7377	0,8599	1,2308	6185	0,24	0,18	0,35	0,23
	AB546335.1	LMMV	Lagenaria mild mosaic virus	1,111	0,8524	0,8686	1,1707	0,6373	1,2477	1,1255	1,0669	1,1505	1,1508	1,0382	0,5948	1,0359	0,7474	0,9971	1,2191	3860	0,29	0,22	0,28	0,22
	AM745758.1	LeVX	Lettuce virus X	1,1301	0,7666	1,0385	1,0014	0,6297	1,2328	1,0198	1,1954	1,0612	1,1176	0,9603	0,8789	1,0943	0,8766	0,9986	1,0048	7212	0,26	0,20	0,34	0,21
	AJ633822.2	LiVX	Lily virus X	0,9451	0,9474	1,1148	0,9583	0,7624	1,0296	0,9661	1,2717	1,1645	1,1053	0,9529	0,7863	1,0655	0,8915	0,97	1,0704	5823	0,25	0,22	0,30	0,23
	DQ660333.1	MMV	Malva mosaic virus	1,0362	0,8866	0,9785	1,1171	0,7135	1,188	0,9339	1,251	1,1864	1,0617	1,0465	0,6001	1,0881	0,8533	1,0596	0,9882	6858	0,29	0,26	0,24	0,21
	AY789138.1	MVX	Mint virus X	1,0733	0,963	1,0133	0,9487	0,516	1,0986	1,1386	1,1352	1,0988	1,0971	0,8681	1,0253	1,1909	0,8129	1,0652	0,8953	5914	0,21	0,20	0,35	0,24
	D13747.1	NMV	Narcissus mosaic virus	1,0879	0,851	1,0184	1,0368	0,6236	1,1944	1,0676	1,1857	1,1669	1,0937	0,8807	0,8178	1,1167	0,8477	1,0495	0,9614	6955	0,28	0,25	0,27	0,21
	AB219105.1	NVX	Nerine virus X	1,0173	0,9398	0,9946	1,0472	0,7557	1,113	0,9774	1,2663	1,1413	1,1027	0,9314	0,7824	1,0345	0,8221	1,1304	0,9541	6582	0,30	0,22	0,29	0,20
	AY366209.1	OVX	Opuntia virus X	1,1137	0,8649	0,9527	1,0425	0,4939	1,1133	1,1806	1,3396	1,1702	1,2827	0,9329	0,5766	1,1158	0,6901	0,9775	1,1764	6653	0,29	0,21	0,29	0,21
	D13957.1	PMV	Papaya mosaic virus	1,1034	0,8733	0,8694	1,1301	0,6163	1,2857	1,0653	1,1626	1,1599	1,0789	1,1859	0,5037	1,0556	0,8063	0,9052	1,2185	6656	0,30	0,22	0,25	0,23
	AF484251.1	PMV	Pepino mosaic virus	1,0977	0,8719	1,0004	1,0101	0,6046	1,177	0,9081	1,5631	1,28	1,1268	0,9675	0,3565	1,0546	0,8136	1,1746	0,9579	6450	0,33	0,27	0,23	0,18
	AB353071.1	PhVX	Phaius virus X	0,9446	0,9597	1,0459	1,0488	0,8701	1,0383	0,8506	1,2859	1,1174	1,1447	1,0778	0,6405	1,0488	0,8438	0,9954	1,094	5816	0,27	0,22	0,27	0,24
	JF930327.1	PiVX	Pitaya virus X	1,0677	0,8661	0,9344	1,1316	0,623	1,0548	1,0734	1,3032	1,1361	1,2852	1,0369	0,518	1,1145	0,7507	0,9616	1,1505	6677	0,27	0,22	0,28	0,23
	Z21647.1	PAMV	Plantago asiatica mosaic virus	1,133	0,7396	0,9738	1,1111	0,6363	1,0823	1,2288	0,928	1,0261	1,1816	0,9842	0,8445	1,0977	0,8945	0,8697	1,2235	6128	0,24	0,17	0,39	0,20
	S73580.1	PAMV	Potato aucuba mosaic virus	1,1087	0,846	0,9344	1,0981	0,679	1,1365	0,9848	1,3115	1,1836	1,164	1,0163	0,5213	1,0414	0,8838	1,0961	0,9813	7059	0,31	0,26	0,22	0,21
	D00344.1	PVX	Potato virus X	0,9722	0,8111	1,018	1,2067	0,6685	1,2101	0,9734	1,2599	1,2795	1,1785	0,9891	0,4613	1,0785	0,8608	1,0142	1,0192	6435	0,31	0,23	0,24	0,23
	D12517.1	SVX	Schlumbergera virus X	1,1238	0,8656	0,9526	1,0649	0,6637	1,0898	1,005	1,2596	1,0452	1,1275	1,0638	0,7423	1,1636	0,8993	0,9706	0,9654	5966	0,25	0,24	0,28	0,23
	AY366207.2	SMYEV	Strawberry mild yellow edge virus	1,1086	0,8595	0,9164	1,1079	0,5227	1,1515	1,1276	1,346	1,1979	1,1815	1,0271	0,4818	1,1324	0,8002	0,9416	1,1112	6633	0,30	0,23	0,26	0,21
	JN389521.1	TRMV	Tamus red mosaic virus	1,1448	0,8308	0,9824	1,0189	0,4815	1,2698	1,1436	1,1884	1,1473	1,1359	0,93	0,7637	1,1919	0,7811	0,9533	1,0418	6494	0,28	0,24	0,25	0,24
	AB066288.1	TVX	Tulip virus X	1,1706	0,6789	0,9969	1,0855	0,6641	1,1104	1,1317	1,0713	1,0077	1,1962	0,9876	0,8363	1,0789	0,9357	0,9093	1,1283	6054	0,25	0,18	0,37	0,20
	MF150240.1	VVX	Vanilla virus X	1,107	0,8733	0,962	1,0419	0,5938	1,2376	1,1217	1,1226	1,1175	1,1067	0,9408	0,8198	1,1043	0,7766	1,0116	1,073	6295	0,28	0,21	0,30	0,21
	X06728.1	WCMV	White clover mosaic virus	1,1321	0,8818	0,9567	1,0152	0,6063	1,1291	1,0616	1,4222	1,1158	1,1339	1,0158	0,5505	1,1775	0,7906	0,9578	1,066	5845	0,30	0,26	0,28	0,16
	KJ711908.1	YVX	Yam virus X	1,0598	0,8204	1,0478	1,0818	0,6492	1,22	1,0032	1,2413	1,172	1,199	0,8907	0,6208	1,1527	0,761	1,0526	1,0121	6158	0,30	0,26	0,24	0,20
	AY366208.1	ZVX	Zygocactus virus X	1,0398	0,8401	0,9444	1,1766	0,561	1,0331	1,1996	1,2068	1,1786	1,2593	0,9425	0,6365	1,1018	0,7956	0,9656	1,1115	6624	0,26	0,20	0,31	0,22

	AA	AT	AC	AG	TA	TT	TC	TG	CA	CT	CC	CG	GA	GT	GC	GG
Average	1,05482	0,86663	1,006	1,0545	0,662008	1,1528	1,04104	1,2271	1,1337	1,14653	0,96577	0,7176	1,09877	0,81963	1,00344	1,055373
Standard deviation	0,05959	0,05959	0,0596	0,0596	0,059587	0,0596	0,05959	0,0596	0,05959	0,05959	0,05959	0,0596	0,05959	0,05959	0,059587	0,059587

		Significance	Values
Underrepresented < 0.78		*	0.78-0.70
		**	0.50-0.70
		***	<0.50
Overrepresented > 1.23		*	1.23-1.3
		**	1.30-1.5
		***	>1.50
