# Supplementary material for: Plant Virus Genome Is Shaped by Specific Dinucleotide Restrictions That Influence Viral Infection
Source: mBio. 2020 Feb 18;11(1):e02818-19. doi: 10.1128/mBio.02818-19 (PMC7029135; doi:10.1128/mBio.02818-19)
Supplement: TABLE S3 [file mBio.02818-19-st003.pdf]

Table S3. Odds ratio for members of the family *Closteroviridae*

Genus	ID	Abrev.	Name	AA	AT	AC	AG	TA	TT	TC	TG	CA	CT	CC	CG	GA	GT	GC	GG	Size	Proporciones nucleótidos A	T	C	G
Ampelovirus	**KC904540.1**	BVBAV	Blackberry vein banding-associated virus	1,0174	0,7744	1,125	1,1133	0,9127	1,096	1,0187	0,9745	0,9587	1,0748	0,9544	0,9969	1,0961	1,0615	0,9001	0,9233	18643	0,26	0,27	0,20	0,28
	**JQ023131.1**	GLAV1	Grapevine leafroll-associated virus 1	1,0944	0,9127	1,0073	0,9923	0,8639	1,149	1,0916	0,9161	0,9218	0,943	0,7999	1,2792	1,1036	0,9699	1,0345	0,9024	18659	0,27	0,28	0,18	0,27
	**AF037268.2**	GLAV3	Grapevine leafroll-associated virus 3	1,0537	0,8382	1,0927	1,0457	0,9498	1,1549	1,0052	0,8854	0,9702	0,9897	0,9234	1,0966	1,0209	1,0083	0,9583	1,0018	17919	0,26	0,28	0,20	0,27
	**FJ467503.1**	GLAV4	Grapevine leafroll-associated virus 4	1,1156	0,8288	1,008	1,0502	0,6692	1,1282	1,12	1,1577	1,108	1,1588	0,9944	0,6948	1,1542	0,9365	0,8587	0,9961	13830	0,29	0,28	0,19	0,24
	**LC052212.1**	GLAV13	Grapevine leafroll-associated virus 13	1,0459	1,0095	0,9213	1,0024	0,8684	1,1343	1,1581	0,8793	1,0217	0,9037	0,8775	1,1807	1,0677	0,9265	1,031	0,971	17607	0,29	0,26	0,21	0,24
	**AF531505.1**	LCV2	Little cherry virus 2	1,136	0,769	1,1424	1,0152	0,7591	1,1304	1,1358	1,0314	1,0156	1,0446	0,981	0,9352	1,1288	1,0911	0,6525	0,9909	15045	0,29	0,30	0,18	0,23
	**AF414119.3**	PiMWAV1	Pineapple mealybug wilt-associated virus 1	1,0703	0,8629	1,0284	1,047	0,7763	1,1694	1,0708	1,0314	1,0298	1,0282	0,986	0,9422	1,1439	0,9556	0,8958	0,9504	13071	0,30	0,27	0,18	0,24
	**AF283103.1**	PiMWAV2	Pineapple mealybug wilt-associated virus 2	0,9719	0,9516	1,1189	0,9998	1,082	1,0279	0,8532	0,9846	0,888	0,9991	0,8791	1,2104	1,0198	1,0228	1,1203	0,8659	14861	0,28	0,29	0,18	0,25
	**DQ399259.2**	PiMWAV3	Pineapple mealybug wilt-associated virus 3	1,0615	0,8589	1,1277	0,9809	0,7893	1,1217	1,0433	1,0935	1,0055	1,021	0,9917	0,9742	1,1655	1,0222	0,7922	0,9323	11870	0,30	0,27	0,19	0,23
	**EF546442.1**	PluBNSPAV	Plum bark necrosis stem pitting-associated virus	1,1541	0,8421	1,044	1,0019	0,6893	1,0775	1,0905	1,1816	1,0894	1,1635	1,0524	0,629	1,1768	0,9451	0,7821	1,0462	14214	0,27	0,32	0,19	0,23
Closterovirus	**U51931.1**	BYSV	Beet yellow stunt virus	1,1474	0,7246	1,1247	1,0301	0,8146	1,1798	1,045	0,9766	0,8564	1,1138	0,8813	1,1443	1,1615	1,0238	0,9039	0,8643	10545	0,28	0,27	0,21	0,24
	**X73476.1**	BYV	Beet yellows virus	1,3538	0,6365	1,1222	0,9525	0,6855	1,2622	1,1052	0,9162	0,7655	1,062	0,8148	1,3453	1,2271	1,0086	0,9168	0,8276	15480	0,25	0,29	0,22	0,24
	**GU234166.1**	CaNFV	Carnation necrotic fleck virus	1,2134	0,75	1,1304	0,947	0,7475	1,2032	1,063	0,974	0,9201	0,9479	0,7978	1,3541	1,1526	1,0819	0,9587	0,7554	10557	0,27	0,29	0,22	0,23
	**FJ869862.1**	CaYLV	Carrot yellow leaf virus	1,313	0,7968	0,9437	0,9425	0,6969	1,2215	1,176	0,8844	0,8531	0,9977	0,9458	1,2481	1,1825	0,9588	0,8961	0,9456	16349	0,27	0,28	0,24	0,21
	**U16304.1**	CiTV	Citrus tristeza virus	1,1579	0,8094	1,1937	0,923	0,8809	1,126	1,03	0,9392	0,8291	0,9484	0,9037	1,3038	1,1267	1,073	0,8538	0,9063	19296	0,24	0,30	0,20	0,25
	**AY881628.1**	GLAV2	Grapevine leafroll-associated virus 2	1,2404	0,8221	0,962	0,9934	0,7535	1,1739	1,0971	0,9702	0,8833	0,9981	0,7921	1,2838	1,1325	0,9818	1,095	0,8131	16494	0,25	0,29	0,21	0,26
	**AY792620.1**	MV1	Mint virus 1	1,1926	0,8466	1,0734	0,9102	0,7223	1,0888	1,2253	0,9713	0,8979	1,0201	0,781	1,2985	1,2337	1,0368	0,8567	0,8411	15450	0,25	0,29	0,23	0,23
	**DQ357218.1**	RMV	Raspberry leaf mottle virus	1,237	0,8076	0,9821	1,0576	0,7862	1,1037	1,1634	0,8791	0,8613	1,065	0,9496	1,1008	1,224	0,9696	0,8467	0,9983	17481	0,22	0,31	0,25	0,22
	**KJ748003.1**	RoLRAV	Rose leaf rosette-associated virus	1,1574	0,8361	1,0711	0,9825	0,7468	1,1047	0,9838	1,1297	0,9869	1,069	1,0165	0,9205	1,15	0,9793	0,9395	0,9328	17656	0,24	0,29	0,21	0,26
	**DQ860839.1**	SCFAV	Strawberry chlorotic fleck-associated virus	1,103	0,8223	1,0382	1,0828	0,8291	1,1141	1,0155	1,0422	1,0076	1,1479	0,9064	0,8663	1,0972	0,9499	1,0052	0,9456	17039	0,28	0,31	0,18	0,23
	**KT203917.1**	TV1	Tobacco virus 1	1,1423	0,7965	1,09	1	0,7563	1,1469	1,1094	1,0138	0,9519	1,0323	0,8748	1,1292	1,1736	1,0346	0,8677	0,8674	15395	0,28	0,29	0,21	0,23
Crinivirus	**NC_010560.1 NC_010561.1**	BYDV	Bean yellow disorder virus	1,0541	0,9906	0,9829	0,9372	0,717	1,0876	1,1061	1,2588	1,2429	0,9093	1,1379	0,619	1,1413	0,9563	0,7599	1,0193	17495	0,34	0,30	0,16	0,20
	**NC_005209.2 NC_005210.2**	BPYV	Beet pseudoyellows virus	1,0999	0,8969	1,0181	0,9864	0,6083	1,1624	1,2503	1,0886	1,0941	0,9974	0,8717	0,9924	1,3136	0,922	0,7488	0,9037	15909	0,30	0,29	0,20	0,21
	**NC_006962.2 NC_006963.2**	BYVAV	Blackberry yellow vein-associated virus	1,0346	0,9063	0,961	1,12	0,7137	1,0776	1,1886	1,1445	1,1549	1,1003	1,0533	0,5883	1,2479	0,9397	0,7358	0,9448	15716	0,30	0,31	0,17	0,21
	**NC_004809.1 NC_004810.1**	CYSDV	Cucurbit yellow stunting disorder virus	1,0538	0,9749	0,9864	0,9619	0,7623	1,0866	1,0911	1,1811	1,1407	0,9396	1,1359	0,7604	1,1483	0,9621	0,7865	0,9834	17099	0,33	0,30	0,16	0,21
	**NC_038786.1 NC_038787.1**	DVCV	Diodia vein chlorosis virus	1,0733	0,9402	0,9585	1,0047	0,7848	1,0861	1,1394	1,1139	1,0352	1,0494	1,1213	0,7705	1,1887	0,9261	0,7566	0,9905	16230	0,33	0,31	0,16	0,20
	**NC_012909.1 NC_012910.1**	LCV	Lettuce chlorosis virus	1,0397	0,9803	0,9868	0,9755	0,7527	1,0486	1,1279	1,2236	1,1755	0,9327	1,1334	0,7059	1,1474	1,0173	0,7294	0,9581	17147	0,33	0,30	0,17	0,21
	**NC_003617.1 NC_003618.1**	LIYV	Lettuce infectious yellows virus	0,9968	0,9551	0,9934	1,0782	0,8382	1,1038	1,1046	1,0482	1,1642	0,9367	1,0649	0,7539	1,1205	0,9731	0,805	0,9799	15311	0,35	0,30	0,15	0,20
	**NC_006061.1 NC_006062.1 NC_006063.1**	PYVV	Potato yellow vein virus	1,0428	0,9579	0,9208	1,0548	0,7291	1,0489	1,2449	1,1818	1,1096	1,0457	1,0631	0,6983	1,2373	0,9617	0,7192	0,8879	17265	0,34	0,30	0,17	0,20
	**NC_005895.2 NC_005896.2**	SPAV	Strawberry pallidosis-associated virus	1,0888	0,972	0,8745	1,013	0,7073	1,1198	1,1701	1,1199	1,035	1,0117	1,1609	0,7947	1,2841	0,8496	0,7965	0,967	16044	0,31	0,31	0,17	0,21
	**NC_004123.1 NC_004124.1**	SPCSV	Sweet potato chlorotic stunt virus	1,0391	0,9172	1,0428	1,0372	0,7817	1,0754	1,125	1,098	1,0439	0,9811	1,1019	0,8895	1,2382	1,0172	0,6785	0,8842	17630	0,30	0,32	0,16	0,22
	**NC_038788.1 NC_038789.1**	TVCV	Tetterwort vein chlorosis virus	1,0747	0,9623	0,9908	0,9506	0,7733	1,0996	1,1115	1,1039	1,0635	0,9542	1,0842	0,9007	1,1682	0,9486	0,7809	1,0027	16580	0,31	0,31	0,17	0,21
	**NC_007340.1 NC_007341.1**	TCV	Tomato chlorosis virus	1,0693	0,9244	1,0706	0,9677	0,7518	1,0943	1,0884	1,1071	1,0768	0,968	1,0525	0,9093	1,2027	0,9856	0,7504	0,9589	16837	0,28	0,32	0,17	0,23
	**NC_013258.1 NC_013259.1**	TICV	Tomato infectious chlorosis virus	1,0204	0,9526	1,0611	0,9853	0,7879	1,0649	1,1081	1,1734	1,1693	0,9287	1,0631	0,7728	1,1281	1,0427	0,7051	0,9588	16184	0,34	0,29	0,16	0,21
Velarivirus	**KR349464.1**	APV1	Areca palm velarivirus 1	1,0688	0,9942	0,9457	0,9352	0,8106	1,0847	1,0654	1,1365	1,0964	0,976	1,0114	0,8514	1,122	0,881	0,9722	1,0155	16080	0,33	0,32	0,17	0,18
	**HM588723.1**	CV1	Cordyline virus 1	1,1057	0,9002	1,0452	0,9206	0,7249	1,1795	1,0582	1,1851	1,1339	0,9432	0,9955	0,8366	1,1384	0,9433	0,8142	0,9902	16883	0,35	0,31	0,16	0,18
	**JQ599282.2**	CV2	Cordyline virus 2	1,1255	0,8939	1,0307	0,9209	0,7243	1,1915	1,0964	1,1052	1,0868	0,9613	0,9427	0,954	1,1576	0,9069	0,8303	1,0086	15107	0,34	0,31	0,16	0,19
	**JQ599283.2**	CV3	Cordyline virus 3	1,1191	0,8859	0,9967	0,9811	0,741	1,2065	1,0819	1,0515	1,0407	0,9663	1,0831	0,9103	1,1985	0,8792	0,7892	1,0217	16274	0,34	0,32	0,16	0,18
	**JQ599284.2**	CV4	Cordyline virus 4	1,0556	0,974	0,9914	0,9528	0,7714	1,1359	1,1346	1,0635	1,1305	0,905	0,9603	0,9609	1,1673	0,903	0,8281	1,011	14618	0,34	0,31	0,16	0,19
	**HE588185.1**	GLAV7	Grapevine leafroll-associated virus 7	1,1056	0,9206	1,1048	0,8743	0,704	1,1246	1,0918	1,2176	1,1486	0,9413	1,035	0,8216	1,173	0,9811	0,6651	1,0007	16403	0,33	0,31	0,15	0,21
	**Y10237.1**	LCV1	Little cherry virus 1	1,0987	0,9456	0,9673	0,9554	0,7347	1,1056	1,089	1,1884	1,0685	1,0673	0,9495	0,8136	1,2271	0,858	0,944	0,9095	16934	0,32	0,33	0,16	0,19
Unknown	**KJ572575.1**	MVBAV	Mint vein banding-associated virus	1,0758	0,8653	1,1626	0,9287	0,8068	1,1999	1,052	0,913	1,0007	0,9567	0,8526	1,2143	1,1807	0,9333	0,8549	1,0037	13387	0,28	0,30	0,21	0,21
	**AB923924.1**	PVB	Persimmon virus B	1,0629	0,8746	1,1153	0,9911	0,9605	1,0729	0,9334	1,0086	0,8939	1,0321	1,0497	1,0457	1,0547	1,0336	0,9094	0,9649	18569	0,28	0,29	0,18	0,24

	AA	AT	AC	AG	TA	TT	TC	TG	CA	CT	CC	CG	GA	GT	GC	GG
Average	1,10428	0,88097	1,0362	0,9908	0,778277	1,1249	1,09441	1,0592	1,02157	1,00541	0,97738	0,9651	1,16103	0,97347	0,849433	0,945181
Standard deviation	0,07857	0,07857	0,0786	0,0551	0,078573	0,0786	0,07857	0,0786	0,07857	0,07857	0,07857	0,0786	0,07857	0,07857	0,078573	0,078573

		Significance	Values
Underrepresented < 0.78		*	0.78-0.70
		**	0.50-0.70
		***	<0.50
Overrepresented > 1.23		*	1.23-1.3
		**	1.30-1.5
		***	>1.50
