# Supplementary material for: Plant Virus Genome Is Shaped by Specific Dinucleotide Restrictions That Influence Viral Infection
Source: mBio. 2020 Feb 18;11(1):e02818-19. doi: 10.1128/mBio.02818-19 (PMC7029135; doi:10.1128/mBio.02818-19)
Supplement: TABLE S4 [file mBio.02818-19-st004.pdf]

Table S4. Odds ratio for members of the family *Bromoviridae*

Genus	ID	Abrev.	Name	AA	AT	AC	AG	TA	TT	TC	TG	CA	CT	CC	CG	GA	GT	GC	GG	Size	A	T	C	G
Alfamovirus	K02703.1/L00163.1/X01572.1	AMV	Alfalfa mosaic virus	1,1276	1,006	0,8314	0,9895	0,7276	1,1052	1,0833	1,1281	0,9647	0,9901	1,148	0,9161	1,2359	0,8615	0,9639	0,9194	8274	0,28	0,29	0,21	0,22
Anulavirus	AB724113.1/AB724114.1/AB724115.1	ALMMV	Amazon lily mild mottle virus	1,2074	0,8602	0,9735	0,983	0,7375	1,0339	1,0709	1,1692	0,9837	1,1318	0,9825	0,8665	1,1251	1,0004	0,9537	0,9093	8206	0,25	0,31	0,19	0,25
	AJ272327.1/AJ272328.2/AJ272329.1	PZSV	Pelargonium zonate spot virus	1,1502	0,8853	0,9721	1,0029	0,6956	1,0765	1,1219	1,1337	0,9938	1,0969	0,9964	0,8934	1,2143	0,9552	0,8859	0,9152	8477	0,26	0,30	0,19	0,25
Bromovirus	M60291.1/M64713.1/M65138.1	BBMV	Broad bean mottle virus	1,1787	0,9137	0,8922	0,9828	0,7424	1,0456	1,11	1,1588	0,9713	1,0228	1,0603	0,9559	1,1272	1,0281	0,9428	0,8625	8250	0,28	0,29	0,20	0,23
	J02042.1/X01678.1/X02380.1	BrMV	Brome mosaic virus	1,0821	0,9059	0,9548	1,0557	0,7592	1,0289	1,07	1,1566	0,9813	1,128	0,992	0,8863	1,1958	0,9571	0,9769	0,8662	8210	0,26	0,28	0,21	0,25
	M28817.1/M28818.1/M65139.1	CoCMV	Cassia yellow blotch virus	1,0938	0,9118	0,9396	1,0452	0,7928	1,0424	1,073	1,1287	0,9259	1,1346	1,0825	0,858	1,2006	0,9424	0,9159	0,9071	8118	0,28	0,29	0,19	0,24
	AB194806.2/AB194807.2/AB194808.1	CYBV	Cowpea chlorotic mottle virus	1,1807	0,8289	0,9022	1,0807	0,6471	1,1112	1,1539	1,1261	0,9778	1,0999	1,1006	0,8262	1,2278	0,9748	0,846	0,9117	7988	0,27	0,28	0,21	0,25
	AB444583.1/AB444584.1/AB444585.1	MYFV	Melandrium yellow fleck virus	1,1063	0,9183	0,96	1,0099	0,8118	1,069	1,0602	1,0913	0,9232	1,0421	1,1307	0,9281	1,1844	0,9739	0,8577	0,9272	8535	0,28	0,30	0,19	0,23
	AB080598.1/AB080599.1/AB080600.1	SBLV	Spring beauty latent virus	1,1577	0,9088	0,8777	1,0244	0,7816	1,0932	1,0783	1,0767	0,8305	1,1108	1,1034	0,9783	1,2234	0,9037	0,9645	0,8895	8363	0,28	0,30	0,18	0,24
Cucumovirus	FM881899.1/FM881900.1/FM881901.1	GMMV	Gayfeather mild mottle virus	1,1123	0,8732	1,068	0,9507	0,7475	1,0452	1,0992	1,1333	0,9668	0,9745	1,0079	1,0611	1,2082	1,118	0,7947	0,8332	8499	0,26	0,27	0,24	0,23
	U15728.1/U15729.1/U15730.1	PSV	Peanut stunt virus	1,0997	0,9157	0,9742	1,0189	0,8182	0,9989	1,0644	1,1347	0,9462	1,0804	1,0939	0,8662	1,1682	1,0124	0,8553	0,9483	8492	0,25	0,28	0,23	0,23
	AJ277268.1/D10044.1/D10663.1	TAV	Tomato aspermy virus	1,0615	0,9937	1,0045	0,9297	0,7973	0,9395	1,1221	1,1977	0,9987	0,9807	1,0301	0,9974	1,1943	1,1061	0,8057	0,8251	8870	0,26	0,29	0,22	0,22
Ilarivirus	JX463340.1/JX463341.1/JX463342.1	AgLV	Ageratum latent virus	1,1115	0,9781	0,969	0,9091	0,7144	1,0874	0,9911	1,266	0,9668	0,9326	1,3302	0,854	1,293	0,9618	0,7692	0,8614	8577	0,29	0,31	0,19	0,22
	AF235033.1/AF235165.1/AF235166.1	AmPLPV	American plum line pattern virus	1,1078	0,9215	1,0109	0,9716	0,6866	1,1742	1,125	1,022	0,9445	0,9208	1,1283	1,0474	1,3074	0,9391	0,7347	0,9635	7830	0,26	0,30	0,20	0,25
	AF174584.1/AF174585.1/U15608.1	ApMV	Apple mosaic virus	0,9735	1,0287	0,9568	1,0301	0,657	1,1949	1,1576	1,068	0,9029	0,8858	1,1556	1,1105	1,4394	0,8527	0,7825	0,8221	8511	0,29	0,27	0,18	0,27
	EU919666.1/EU919667.1/X86352.1	AV2	Asparagus virus 2	1,1051	1,0393	0,8771	0,9341	0,6876	1,0786	0,9864	1,2693	1,0348	0,934	1,3004	0,7891	1,2516	0,9109	0,9039	0,9069	8653	0,27	0,30	0,20	0,23
	DQ091193.1/DQ091194.2/DQ091195.2	BCRV	Blackberry chlorotic ringspot virus	1,1142	1,0203	0,949	0,8816	0,6746	1,0989	1,008	1,2565	0,9979	0,858	1,2492	0,97	1,2792	0,9707	0,8405	0,8395	8647	0,28	0,30	0,20	0,23
	KF031037.1/KF031038.1/KF031039.1	BSV	Blueberry shock virus	1,0016	0,9644	1,0129	1,0292	0,7743	1,1679	1,0699	1,0104	0,8623	0,9181	1,2028	1,1044	1,3644	0,9048	0,753	0,8774	7989	0,28	0,29	0,18	0,25
	U17390.1/U17726.1/U23715.1	CLRV	Citrus leaf rugose virus	1,0511	1,0772	0,8496	0,9778	0,7206	1,0839	1,0091	1,2048	1,0515	0,866	1,2305	0,9051	1,2471	0,9253	0,9576	0,8505	8683	0,27	0,29	0,21	0,23
	EF584664.1/EF584665.1/U17389.1	CVV	Citrus variegation virus	1,1346	1,0334	0,8687	0,9142	0,6698	1,1019	1,0315	1,2272	0,9733	0,9171	1,2524	0,9174	1,2902	0,9024	0,8951	0,8781	8656	0,27	0,30	0,20	0,23
	U34050.1/U57047.1/U85399.1	EMV	Elm mottle virus	1,0728	1,0262	0,8853	0,9873	0,6726	1,0995	1,0212	1,2276	1,0283	0,9039	1,2874	0,8318	1,2961	0,9334	0,8507	0,8809	8620	0,27	0,29	0,21	0,24
	AY682102.1/AY707771.1/AY707772.1	FCLV	Fragaria chiloensis latent virus	1,085	0,9846	0,9641	0,959	0,7039	1,0869	1,0244	1,1783	1,0547	0,8961	1,2149	0,914	1,2689	0,9796	0,8392	0,8671	8485	0,25	0,32	0,19	0,24
	AY500236.1/AY500237.1/AY500238.1	HJLV	Humulus japonicus latent virus	1,0973	1,0557	0,7579	1,01	0,7671	1,0877	0,9919	1,1893	0,9758	1,0007	1,2896	0,7862	1,1913	0,8199	1,0733	0,9258	8130	0,29	0,29	0,19	0,23
	FN669168.1/FN669169.1/HE572565.1	LLCV	Lilac leaf chlorosis virus	1,0425	0,8915	1,0658	1,0215	0,6448	1,2421	1,1129	1,0414	0,9474	0,9223	1,065	1,0928	1,3923	0,9141	0,7451	0,8557	8283	0,28	0,28	0,20	0,25
	AY496068.1/AY496069.1/U35145.1	PMV	Parietaria mottle virus	1,0734	1,0061	1,0006	0,9144	0,6452	1,1058	1,06	1,2096	0,9857	0,8655	1,1611	1,0348	1,3401	0,9837	0,7983	0,8214	8708	0,27	0,28	0,20	0,25
	KT290039.1/KT290040.1/KT290041.1	PriRV	Privet ringspot virus	1,0735	1,0156	0,9648	0,9187	0,6145	1,1051	1,0752	1,2768	1,1231	0,8602	1,1394	0,9064	1,3135	0,9647	0,8206	0,8138	8418	0,28	0,30	0,20	0,22
	AF277662.1/L28145.1/U57648.1	PruDV	Prune dwarf virus	1,1318	0,9311	0,985	0,9413	0,6753	1,109	1,0026	1,2622	1,025	1,0069	1,2779	0,7196	1,2723	0,9282	0,7804	0,9376	8096	0,28	0,31	0,19	0,22
	AF278534.1/AF278535.1/U57046.1	PruNRV	Prunus necrotic ringspot virus	0,9826	0,9614	1,0362	1,0354	0,7665	1,1206	1,0351	1,0917	0,9124	0,8829	1,2007	1,0723	1,3486	0,9957	0,7693	0,8021	7880	0,27	0,28	0,19	0,25
	U93192.1/U93193.1/U93194.1	SLV	Spinach latent virus	1,0841	1,0938	0,8596	0,8996	0,7211	1,0484	0,9674	1,2845	1,0102	0,9188	1,3038	0,8449	1,2449	0,8978	0,9587	0,8816	8688	0,27	0,30	0,19	0,24
	AY363228.2/AY743591.2/DQ318818.3	SNSV	Strawberry necrotic shock virus	1,0525	1,0641	0,9705	0,8753	0,6632	1,0654	1,0172	1,3127	1,0731	0,8687	1,2439	0,8833	1,3229	0,9424	0,8149	0,832	8550	0,28	0,30	0,19	0,23
	KT779204.1/KT779205.1/KT779206.1	TNSV	Tomato necrotic streak virus	1,0506	1,1037	0,8709	0,9286	0,722	1,0717	0,9935	1,2466	0,9953	0,8833	1,3018	0,8786	1,2852	0,8922	0,893	0,8908	8621	0,28	0,29	0,21	0,23
	U75538.1/U80934.1/X00435.1	TuAMV	Tulare apple mosaic virus	1,1199	1,0533	0,8711	0,9147	0,6837	1,0933	0,9988	1,2339	1,0317	0,8972	1,2566	0,8658	1,2378	0,9151	0,9179	0,917	8632	0,26	0,30	0,21	0,24
	AF226160.1/AF226161.1/AF226162.1	TSV	Tobacco streak virus	1,0997	1,0002	0,9503	0,9234	0,6485	1,1122	1,0604	1,2331	1,0094	0,8764	1,1956	0,9676	1,3109	0,9696	0,8117	0,8284	8622	0,28	0,29	0,20	0,23
Oleavirus	X76993.1/X94346.1/X94347.1	OLV	Olive latent virus 2	1,1472	0,8575	0,9547	1,0496	0,6882	1,1175	1,0912	1,0925	0,9076	1,0471	1,1404	0,9178	1,2655	0,9714	0,83	0,9249	8301	0,25	0,27	0,22	0,26

	AA	AT	AC	AG	TA	TT	TC	TG	CA	CT	CC	CG	GA	GT	GC	GG
Average	1,09619	0,97145	0,9406	0,9735	0,713532	1,0895	1,05699	1,1717	0,97875	0,96632	1,16635	0,9249	1,26082	0,95027	0,861841	0,879212
Standard deviation	0,05226	0,05226	0,0523	0,0523	0,052264	0,0523	0,05226	0,0523	0,05226	0,05226	0,05226	0,0523	0,05226	0,05226	0,052264	0,052264

		Significance	Values
Underrepresented < 0.78		*	0.78-0.70
		**	0.50-0.70
		***	<0.50
Overrepresented > 1.23		*	1.23-1.3
		**	1.30-1.5
		***	>1.50
