# Supplementary figures and images for: Plant Virus Genome Is Shaped by Specific Dinucleotide Restrictions That Influence Viral Infection
Source: mBio. 2020 Feb 18;11(1):e02818-19. doi: 10.1128/mBio.02818-19 (PMC7029135; doi:10.1128/mBio.02818-19)

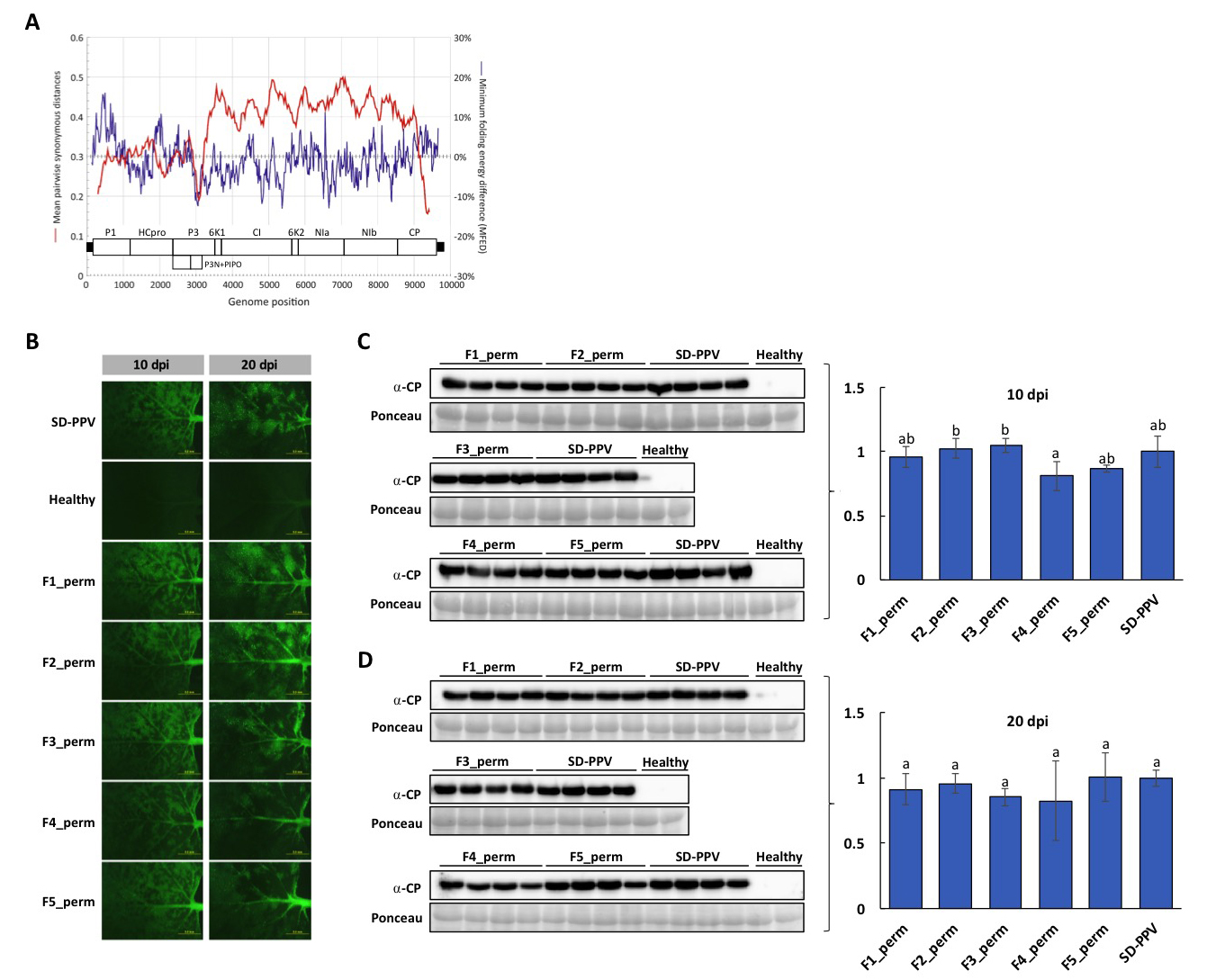

Supplement: FIG S1 [file mBio.02818-19-sf001.jpg]

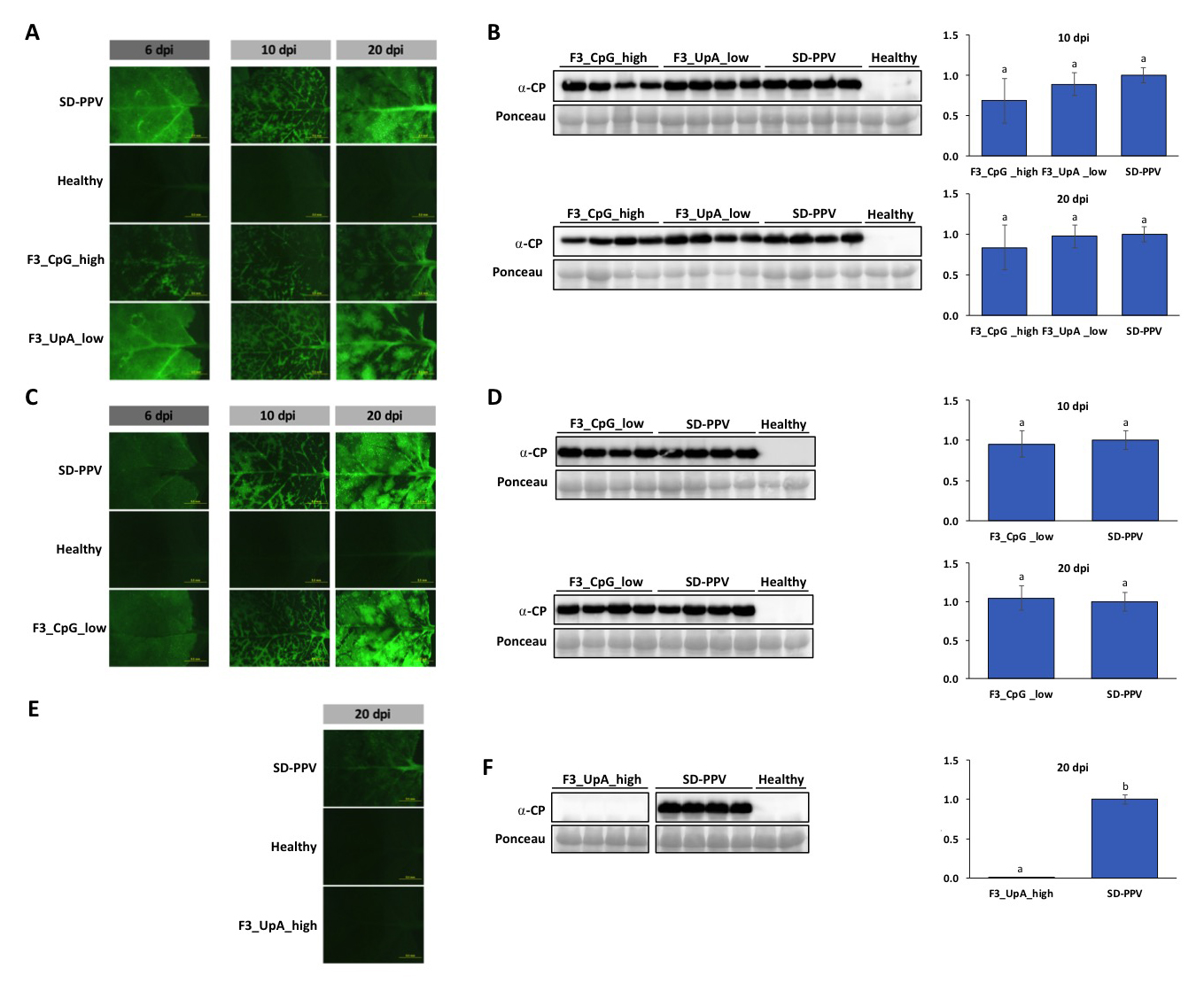

Supplement: FIG S2 [file mBio.02818-19-sf002.jpg]

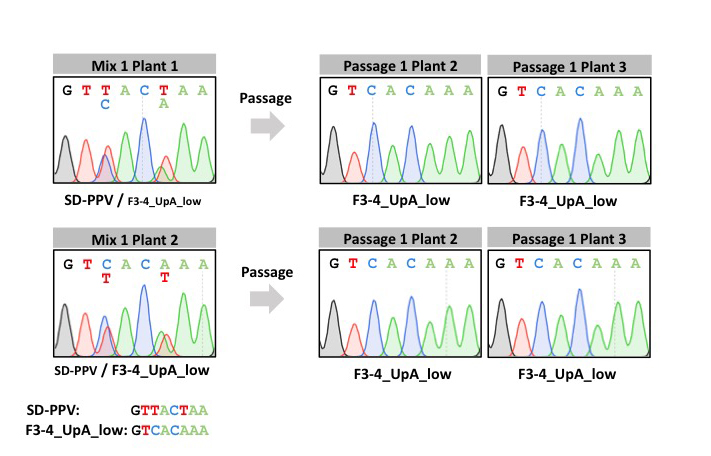

Supplement: FIG S3 [file mBio.02818-19-sf003.jpg]

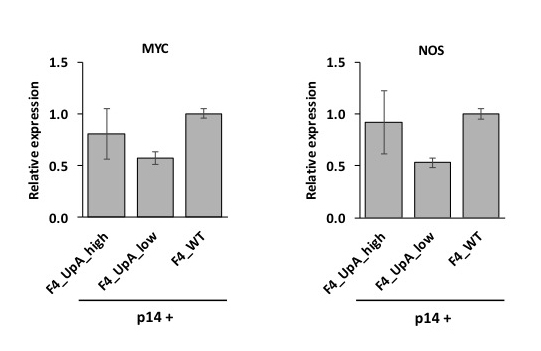

Supplement: FIG S4 [file mBio.02818-19-sf004.jpg]
