# Supplementary material for: Plant Virus Genome Is Shaped by Specific Dinucleotide Restrictions That Influence Viral Infection
Source: mBio. 2020 Feb 18;11(1):e02818-19. doi: 10.1128/mBio.02818-19 (PMC7029135; doi:10.1128/mBio.02818-19)
Supplement: TABLE S5 [file mBio.02818-19-st005.pdf]

Table S5: composition of different PPV-derived fragments

Region	Sequence	Size (bp)	G+C content	Total changes	Sequence identity	CpG freq	CpG total (change)	CpG O/E ratio	UpA freq	UpA total (change)	UpA O/E ratio	ApU freq	ApU total (change)	ApU O/E ratio	Codon usage	
															CAI	Enc_o
**F1**	**Native**	966	44.6%	-	-	0.031	30 (-)	0.626	0.031	30 (-)	0.409	0.080	77 (-)	1.050	0.750	53.44
	**Permuted**	966	44.5%	116	88.0%	0.031	30 (0)	0.629	0.032	31 (+1)	0.420	0.090	76 (-1)	1.031	0.754	55.61
**F2**	**Native**	930	44.1%	-	-	0.043	40 (-)	0.888	0.041	38 (-)	0.528	0.064	59 (-)	0.820	0.731	55.77
	**Permuted**	930	44.2%	113	87.8%	0.043	40 (0)	0.883	0.040	37 (-1)	0.517	0.065	60 (+1)	0.838	0.733	55.37
**F3**	**Native**	1233	44.0%	-	-	0.033	40 (-)	0.669	0.036	44 (-)	0.470	0.073	90 (-)	0.960	0.754	53.94
	**Permuted**	1233	44.2%	150	87.8%	0.033	40 (0)	0.669	0.036	44 (0)	0.470	0.073	90 (0)	0.961	0.750	55.47
	**CpG_low**	1233	42.2%	51	95.7%	0.000	0 (-40)	0.000	0.036	44 (0)	0.435	0.080	99 (+9)	0.978	0.792	46.18
	**UpA_low**	1233	46.5%	32	97.4%	0.033	40 (0)	0.608	0.010	12 (-32)	0.139	0.067	82 (-8)	0.950	0.748	48.91
	**CpG_high**	1233	53.3%	127	89.7%	0.136	167 (+127)	1.922	0.036	44 (0)	0.666	0.060	74 (-16)	1.121	0.608	41.06
	**UpA_high**	1233	38.9%	113	90.8%	0.033	40 (0)	0.888	0.127	157 (+113)	1.373	0.092	113 (+23)	0.988	0.744	43.95
**F4**	**Native**	975	43.0%	-	-	0.027	26 (-)	0.591	0.045	44 (-)	0.556	0.067	65 (-)	0.822	0.776	52.31
	**Permuted**	975	42.7%	128	86.9%	0.027	26 (0)	0.591	0.045	44 (0)	0.556	0.068	65 (0)	0.822	0.773	53.65
	**CpG_low**	975	40.8%	30	96.9%	0.001	1 (-25)	0.025	0.045	44 (0)	0.516	0.073	71 (+6)	0.833	0.806	42.42
	**UpA_low**	975	45.6%	32	96.7%	0.027	26 (0)	0.521	0.012	12 (-32)	0.168	0.060	58 (-7)	0.811	0.764	49.12
	**CpG_high**	975	51.6%	97	90.1%	0.126	123 (+97)	1.919	0.045	44 (0)	0.777	0.051	50 (-15)	0.883	0.606	42.96
	**UpA_high**	975	37.9%	79	91.9%	0.027	26 (0)	0.767	0.126	123 (+79)	1.315	0.093	91 (+26)	0.973	0.758	43.54
	**UpA_high_M_ fixed**	975	42.7%	121	87.6%	0.027	26 (0)	0.591	0.107	104 (+60)	1.315	0.076	74 (+9)	0.935	0.722	49.67
	**U+A_high_UpA_ fixed**	975	38.0%	136	86.1%	0.021	20 (-6)	0.586	0.045	44 (0)	0.047	0.092	90 (+25)	0.966	0.792	44.83
	**ApU_high_ UpA_ fixed**	975	40.4%	49	95.0%	0.021	20 (-6)	0.520	0.045	44 (0)	0.509	0.108	104 (+39)	1.215	0.800	43.52
**F4.1**	**Native**	459	43.4%	-	-	0.024	11 (-)	0.511	0.052	24 (-)	0.662	0.066	30 (-)	0.826	0.828	50.61
	**UpA_high**	459	39.0%	34	92.6%	0.024	11 (0)	0.633	0.127	58 (+34)	1.382	0.094	43 (+13)	1.024	0.755	48.14
**F4.2**	**Native**	516	42.3%	-	-	0.029	15 (-)	0.683	0.039	20 (-)	0.468	0.066	34 (-)	0.795	0.779	55.89
	**UpA_high**	516	36.7%	45	91.3%	0.029	15 (0)	0.926	0.126	65 (+45)	1.263	0.091	47 (+17)	0.914	0.760	45.77
**F5**	**Native**	828	42.5%	-	-	0.028	23 (-)	0.617	0.051	42 (-)	0.616	0.088	73 (-)	1.070	0.782	51.12
	**Permuted**	828	42.5%	100	87.9%	0.028	23 (0)	0.617	0.051	42 (0)	0.616	0.088	73 (0)	1.070	0.733	51.89
