# Supplementary material for: Plant Virus Genome Is Shaped by Specific Dinucleotide Restrictions That Influence Viral Infection
Source: mBio. 2020 Feb 18;11(1):e02818-19. doi: 10.1128/mBio.02818-19 (PMC7029135; doi:10.1128/mBio.02818-19)
Supplement: TABLE S6 [file mBio.02818-19-st006.pdf]

Table S6: list of primers used in this study

Name	Sequence (5’-3’)
**2806**	GACCCTGATGTTGATGTTCGCT
**2807**	GAGGGATTTGAAGAGAGATTTC
**3340**	TCACAAGCAAATTTGGAACG
**3341**	GCATTAATGTTGCGTCACGA
**3344**	AGCGCAACATATCCAGTTGA
**3345**	CACCAATGTCCCATTCTTCC
**3364**	CGAAGTCCACAACCACATCA
**3365**	TGATGTGGTTGTGGACTTCG
**3392**	GAGTGCTCTAAATAAGGACGC
**3393**	GCGTCCTTATTTAGAGCACTC
**3463**	GCAGCTTTCCCTGACAACTT
**3464**	CCACAGAGTCCGTTTAC
**3465**	AGCACTCTTTTGGTATGC
**3466**	CAATTTGAACGTGCTG
**3467**	TCTACCTTCTCTTTAGGGC
**3513**	GGGACAAGTTTGTACAAAAAAGCAGGCTTACCCATCGTGAGCACTCGAGAT
**3514**	GGGGACCACTTTGTACAAGAAAGCTGGGTCTCAGTAAATCCATCCTTCTGGCAG
**3519**	AGACTTGAACGGACTCGAC
**3520**	AGTGCTCACGATGGGTAAG
**3527**	TAGAGCTCGAATTTCCCC
**3528**	ACCCATCTCATAAATAACGTC
