# Supplementary material for: Plant Virus Genome Is Shaped by Specific Dinucleotide Restrictions That Influence Viral Infection
Source: mBio. 2020 Feb 18;11(1):e02818-19. doi: 10.1128/mBio.02818-19 (PMC7029135; doi:10.1128/mBio.02818-19)
Supplement: TABLE S1 [file mBio.02818-19-st001.pdf]

Table S1. Odds ratio for members of the family *Potyviridae*

Genus	Reference	Abbreviation	Name	AA	AT	AC	AG	TA	TT	TC	TG	CA	CT	CC	CG	GA	GT	GC	GG	Size	A	T	C	G
**Arepavirus**	**MH395371.1**	ANRSV	Areca palm necrotic virus	0,992	0,927	1,038	1,071	0,614	1,337	0,943	1,258	1,519	0,904	0,905	0,358	1,083	0,789	1,078	1,059	9434	0,353	0,262	0,165	0,221
**Bevemovirus**	**KY491536.1**	BeVMV	Bellflower veinal mottle virus	1,058	1,045	0,987	0,895	0,544	1,143	1,045	1,339	1,482	0,866	0,769	0,781	1,008	0,907	1,168	0,946	8259	0,284	0,263	0,211	0,242
**Brambiviruses**	**AY994084.1**	BIVY	Blackberry virus Y	0,929	1,029	1,134	0,945	0,731	1,180	0,854	1,303	1,393	0,788	0,862	0,819	1,035	0,957	1,101	0,909	10851	0,314	0,250	0,207	0,229
**Bymoviruses**	**Y10973.1/X90904.1**	BaMMV	Barley mild mosaic virus	1,045	0,908	1,075	0,951	0,561	1,171	1,057	1,296	1,269	1,052	0,761	0,900	1,085	0,870	1,137	0,869	10785	0,275	0,235	0,268	0,222
	**AJ132268.1/AJ132269.1**	BaYMV	Barley yellow mosaic virus	1,087	0,925	0,997	0,986	0,571	1,162	1,041	1,294	1,313	1,065	0,773	0,784	1,060	0,830	1,205	0,903	11219	0,274	0,263	0,242	0,221
	**AJ306718.1/AJ306719.1**	OMV	Oat mosaic virus	1,129	0,931	0,988	0,929	0,608	1,161	0,999	1,319	1,236	1,061	0,867	0,779	1,017	0,829	1,173	0,977	9834	0,278	0,253	0,253	0,216
	**LC055681.1/LC060925.1**	RNMV	Rice necrosis mosaic virus	1,092	0,920	1,031	0,936	0,568	1,208	1,111	1,237	1,259	1,018	0,795	0,841	1,093	0,864	1,040	0,984	10757	0,301	0,248	0,224	0,227
	**MH645041.1/MH645043.1**	WSSMV	Wheat spindle streak mosaic virus	1,091	0,955	0,996	0,949	0,601	1,178	1,038	1,221	1,303	1,007	0,791	0,857	1,046	0,832	1,184	0,950	11854	0,266	0,270	0,243	0,221
	**FJ361768.1/FJ361769.1**	WYMV	Wheat yellow mosaic virus	1,046	0,890	0,986	1,097	0,620	1,202	1,081	1,123	1,203	1,058	0,850	0,873	1,137	0,815	1,118	0,898	7300	0,261	0,255	0,276	0,208
**Ipomovirus**	**GU563327.1**	CBSV	Cassava brown streak virus	1,042	0,949	0,937	1,043	0,618	1,146	1,073	1,279	1,271	0,944	1,025	0,700	1,210	0,935	0,984	0,815	8995	0,318	0,281	0,153	0,249
	**KU935732.1**	CocMoV	Coccinia mottle virus	1,078	0,942	0,869	1,041	0,520	1,233	1,209	1,268	1,310	0,910	0,999	0,677	1,225	0,881	0,950	0,855	9778	0,333	0,268	0,155	0,243
	**AY578085.1**	CVYV	Cucumber vein yellowing virus	1,075	0,961	0,939	0,984	0,580	1,219	1,081	1,265	1,233	0,898	1,007	0,799	1,188	0,889	0,990	0,877	9751	0,324	0,262	0,167	0,247
	**HQ840786.2**	EMLMV	Eggplant mild leaf mottle virus	1,052	0,899	0,935	1,089	0,617	1,182	1,013	1,243	1,351	0,988	0,951	0,641	1,096	0,936	1,094	0,892	9280	0,297	0,268	0,177	0,259
	**EU259611.1**	SqVYV	Squash vein yellowing virus	1,099	0,993	0,885	0,959	0,561	1,183	1,131	1,287	1,231	0,984	0,942	0,750	1,191	0,816	1,050	0,915	9836	0,320	0,268	0,173	0,240
	**Z73124.1**	SPMMV	Sweet potato mild mottle virus	1,089	0,988	0,970	0,923	0,600	1,223	0,938	1,308	1,355	0,891	0,845	0,784	1,054	0,856	1,219	0,926	10818	0,309	0,264	0,181	0,246
	**HE600072.1**	ToMMV	Tomato mild mottle virus	1,103	0,925	0,905	1,025	0,592	1,199	0,984	1,263	1,355	0,947	0,997	0,653	1,086	0,908	1,128	0,917	9281	0,296	0,277	0,168	0,260
	**NC_014791.1**	UCBSV	Ugandan cassava brown streak virus	1,012	0,974	0,878	1,087	0,645	1,162	1,071	1,238	1,369	0,914	1,053	0,579	1,189	0,893	1,047	0,849	9070	0,324	0,290	0,143	0,244
**Macluravirus**	**MG978107.1**	AlpMV	Alpinia oxyphylla mosaic virus	1,079	0,936	1,097	0,871	0,513	1,300	0,961	1,422	1,488	0,886	0,665	0,668	1,072	0,814	1,195	0,952	8213	0,333	0,272	0,186	0,209
	**KP405232.1**	ArLV	Artichoke latent virus	1,070	0,996	1,046	0,877	0,536	1,135	1,035	1,410	1,352	0,984	0,748	0,801	1,071	0,883	1,128	0,916	8287	0,301	0,244	0,213	0,242
	**KU053507.1**	BLDVA	Broad-leafed dock virus A	1,077	0,932	0,977	0,990	0,526	1,230	0,944	1,413	1,391	1,001	0,882	0,607	1,042	0,856	1,184	0,938	8163	0,309	0,241	0,207	0,243
	**MF622947.1**	CaMV	Cardamom mosaic virus	1,050	0,916	1,105	0,937	0,586	1,314	0,890	1,318	1,459	0,890	0,746	0,686	1,037	0,829	1,207	0,976	8249	0,320	0,271	0,193	0,216
	**AB710145.1**	ChYNMV	Chinise yam necrotic mosaic virus	1,112	0,932	1,061	0,868	0,468	1,270	1,007	1,431	1,437	0,923	0,687	0,750	1,073	0,851	1,182	0,907	8224	0,317	0,261	0,198	0,224
	**KX979913.1**	NaLV	Narcissus latent virus	1,069	0,964	1,021	0,934	0,550	1,261	0,950	1,337	1,378	0,925	0,807	0,773	1,053	0,837	1,197	0,930	8177	0,298	0,252	0,213	0,238
	**MF072318.1**	YCNMV	Yam chlorotic necrosis mosaic virus	1,118	0,981	1,026	0,833	0,466	1,222	1,027	1,475	1,496	0,881	0,683	0,699	1,065	0,855	1,198	0,915	8199	0,320	0,271	0,187	0,222
**Poacevirus**	**JX156425.1**	CaVA	Caladenia virus A	1,051	0,910	1,077	0,969	0,723	1,249	0,872	1,187	1,310	0,849	0,856	0,892	0,989	0,962	1,167	0,914	9839	0,304	0,268	0,198	0,229
	**GQ388116.1**	SSMV	Sugarcane streak mosaic virus	0,976	1,020	1,109	0,911	0,664	1,214	1,001	1,227	1,300	0,910	0,768	0,898	1,135	0,813	1,063	0,963	9781	0,314	0,254	0,207	0,225
	**FJ263671.1**	TriMV	Triticum mosaic virus	1,015	0,917	1,135	0,976	0,767	1,202	0,847	1,176	1,261	0,865	0,836	0,948	1,060	0,969	1,130	0,862	10282	0,305	0,280	0,178	0,237
**Potyvirus**	**MF997470.1**	AfEMV	African eggplant mosaic virus	1,006	0,980	0,984	1,027	0,604	1,108	1,072	1,360	1,427	1,046	0,756	0,570	1,078	0,872	1,143	0,919	9646	0,317	0,257	0,191	0,234
	**EU410442.1**	AlWMV	Algerian watermelon mosaic virus	1,059	0,972	0,966	0,978	0,619	1,209	1,064	1,226	1,328	0,962	0,819	0,750	1,074	0,837	1,125	0,983	9795	0,314	0,262	0,191	0,233
	**HM363516.1**	ApVY	Apium virus Y	1,009	0,972	1,079	0,955	0,652	1,135	1,048	1,330	1,336	0,932	0,797	0,736	1,115	0,942	0,991	0,902	9917	0,337	0,258	0,182	0,223
	**DQ925486.2**	ArMV	Arracacha mottle virus	0,979	1,040	0,947	1,023	0,673	1,156	1,039	1,250	1,399	0,907	0,765	0,720	1,120	0,830	1,203	0,880	9630	0,326	0,272	0,170	0,231
	**KJ830760.1**	AsV1	Asparagus virus 1	1,013	0,940	1,096	0,972	0,557	1,247	1,081	1,301	1,432	0,915	0,717	0,693	1,141	0,868	1,002	0,946	9741	0,330	0,263	0,188	0,219
	**DQ851496.1**	BaBrMV	Banana bract mosaic virus	0,996	1,062	1,029	0,910	0,725	1,105	0,965	1,275	1,340	0,893	0,810	0,817	1,059	0,879	1,152	0,943	9711	0,313	0,272	0,183	0,232
	**KU685505.1**	BaVY	Barbacena virus Y	1,013	0,960	0,996	1,031	0,650	1,129	1,000	1,340	1,406	0,959	0,864	0,591	1,059	0,941	1,114	0,894	9837	0,321	0,265	0,184	0,230
	**NC_009741.1**	BaRMV	Basella rugose mosaic virus	0,999	0,979	0,960	1,057	0,648	1,166	0,994	1,300	1,378	1,020	0,863	0,565	1,105	0,828	1,169	0,920	9804	0,322	0,264	0,180	0,234
	**AY864314.2**	BeCMNV	Bean common mosaic necrotic virus	1,044	0,918	1,008	1,024	0,594	1,190	0,973	1,363	1,384	1,053	0,848	0,541	1,083	0,863	1,138	0,931	9893	0,320	0,258	0,185	0,237
	**AJ312437.1**	BeCMV	Bean common mosaic virus	1,002	0,948	1,020	1,039	0,556	1,188	1,031	1,381	1,500	0,990	0,825	0,465	1,086	0,881	1,074	0,953	9992	0,325	0,253	0,182	0,240
	**NC_003492.1**	BeYMV	Bean yellow mosaic virus	1,020	0,920	1,005	1,057	0,605	1,245	0,974	1,298	1,412	0,967	0,912	0,529	1,098	0,864	1,089	0,946	9532	0,327	0,261	0,178	0,235
	**KY012334.1**	BtMV	Beet mosaic virus	0,977	0,970	0,990	1,071	0,562	1,197	1,101	1,276	1,393	0,995	0,781	0,681	1,154	0,840	1,093	0,891	9591	0,310	0,249	0,201	0,240
	**KF649336.1**	BiMV	Bidens mosaic virus	1,038	0,907	1,093	0,979	0,623	1,251	1,031	1,221	1,370	0,909	0,663	0,851	1,085	0,914	1,104	0,895	9556	0,324	0,265	0,184	0,227
	**AF538686.2**	BiMotV	Bidens mottle virus	1,048	0,927	1,055	0,964	0,628	1,204	1,070	1,247	1,349	0,943	0,750	0,778	1,052	0,917	1,065	0,963	9741	0,324	0,260	0,199	0,217
	**JQ807999.1**	BluSVA	Blue squill virus A	1,022	0,947	1,086	0,966	0,553	1,189	1,028	1,336	1,486	0,963	0,682	0,669	1,084	0,896	1,096	0,933	9842	0,309	0,259	0,181	0,250
	**JX867236.1**	BruMV	Brugmasia mosaic virus	0,997	0,963	0,985	1,057	0,645	1,135	1,046	1,310	1,376	1,063	0,844	0,518	1,125	0,850	1,088	0,930	9796	0,326	0,266	0,176	0,232
	**AB551370.1**	BsMoV	Brugmansia suaveolens mottle virus	1,022	1,012	1,018	0,936	0,628	1,119	1,030	1,414	1,379	0,986	0,994	0,420	1,098	0,848	0,938	1,087	9868	0,338	0,264	0,183	0,215
	**EF105297.1**	CaLLV	Calla lily latent virus	1,032	0,956	0,969	1,028	0,625	1,206	0,981	1,280	1,451	0,972	0,833	0,564	1,038	0,855	1,184	0,975	9731	0,317	0,264	0,176	0,243
	**KX013584.1**	CaMV	Callistephus mottle virus	0,984	0,986	1,099	0,954	0,722	1,089	0,960	1,341	1,383	0,903	0,792	0,728	1,011	1,002	1,083	0,910	9859	0,327	0,258	0,193	0,222
	**GQ421689.2**	CaYSV	Canna yellow streak virus	1,030	0,983	1,102	0,889	0,639	1,217	0,939	1,344	1,354	0,836	0,733	0,885	1,061	0,922	1,145	0,876	9501	0,333	0,253	0,190	0,224
	**JX156434.1**	CaTLV	Carrot thin leaf virus	0,980	0,950	1,031	1,060	0,645	1,204	1,015	1,287	1,402	0,951	0,816	0,610	1,097	0,889	1,086	0,909	9491	0,337	0,251	0,183	0,229
	**KP343681.1**	CatMV	Catharanthus mosaic virus	0,985	0,934	1,082	1,038	0,698	1,158	0,979	1,293	1,385	0,981	0,804	0,587	1,073	0,927	1,061	0,926	9575	0,337	0,265	0,180	0,218
	**HQ676607.1**	CeMV	Celery mosaic virus	1,019	0,954	0,987	1,034	0,653	1,195	1,035	1,260	1,418	0,896	0,851	0,624	1,038	0,929	1,096	0,950	9999	0,334	0,258	0,178	0,230
	**JN008909.1**	ChiRSV	Chilli ringspot virus	1,008	0,939	0,980	1,075	0,660	1,124	1,065	1,299	1,396	0,992	0,834	0,578	1,035	0,953	1,100	0,916	9595	0,326	0,256	0,195	0,223
	**AJ237843.3**	ChVMV	Chilli veinal mottle virus	0,992	1,007	0,950	1,043	0,623	1,148	1,069	1,290	1,432	0,872	0,885	0,651	1,092	0,924	1,085	0,892	9711	0,315	0,267	0,189	0,229
	**AB011819.1**	CloYVV	Clover yellow vein virus	1,005	0,906	0,969	1,123	0,579	1,215	1,048	1,331	1,529	0,937	0,895	0,387	1,052	0,945	1,074	0,928	9584	0,333	0,257	0,179	0,231
	**AF499738.1**	CocSV	Cocksfoot streak virus	1,026	1,003	0,992	0,971	0,608	1,133	1,114	1,253	1,270	0,958	0,804	0,885	1,129	0,895	1,076	0,876	9663	0,298	0,249	0,221	0,232
	**JQ801448.1**	ColDV	Colombian datura virus	1,012	0,959	1,036	1,005	0,580	1,152	1,011	1,414	1,493	0,991	0,762	0,489	1,102	0,878	1,130	0,895	9621	0,322	0,276	0,181	0,221
	**AF348210.1**	CowABMV	Cowpea aphid-borne mosaic virus	1,023	0,912	0,972	1,088	0,578	1,210	1,066	1,310	1,421	1,061	0,866	0,450	1,088	0,845	1,076	0,985	9465	0,327	0,253	0,188	0,232
	**KY657266.1**	CVBV	Cucurbit vein banding virus	1,046	1,000	0,991	0,945	0,634	1,152	1,060	1,273	1,336	0,891	0,858	0,785	1,097	0,908	1,060	0,926	9968	0,311	0,274	0,191	0,224
	**JQ723475.1**	CyrEVA	Cyrtanthus elatus virus A	0,990	0,981	1,097	0,964	0,642	1,178	1,029	1,268	1,321	0,895	0,758	0,860	1,163	0,913	1,014	0,863	9908	0,323	0,259	0,177	0,241
	**DQ299908.1**	DapMV	Daphne mosaic virus	1,010	1,021	0,972	0,988	0,594	1,234	1,056	1,234	1,323	0,892	0,720	0,925	1,132	0,828	1,205	0,842	9548	0,311	0,245	0,198	0,246
	**KU556609.1**	DapVY	Daphne virus Y	1,027	0,983	0,979	1,001	0,601	1,230	1,055	1,197	1,271	0,946	0,705	0,957	1,148	0,843	1,185	0,847	9448	0,299	0,247	0,189	0,265
	**AJ298033.1**	DasMV	Dasheen mosaic virus	1,067	0,961	0,970	0,974	0,726	1,156	0,939	1,259	1,293	0,971	0,960	0,662	0,956	0,908	1,142	1,041	10038	0,319	0,253	0,197	0,231
	**JX156422.1**	DoOVA	Donkey orchid virus A	1,050	0,947	1,115	0,922	0,611	1,181	0,981	1,296	1,361	0,933	0,807	0,748	1,148	0,901	1,007	0,923	9868	0,309	0,284	0,164	0,243
	**AB246773.1**	EaAPV	East asian passiflora virus	1,057	0,948	0,984	0,996	0,553	1,190	1,015	1,370	1,443	0,988	0,899	0,501	1,087	0,866	1,083	0,972	10046	0,316	0,266	0,181	0,237
	**KU941946.1**	EndNMV	Endive necrotic mosaic virus	1,005	1,056	1,073	0,872	0,598	1,186	1,037	1,352	1,397	0,851	0,725	0,838	1,009	0,882	1,116	0,994	10000	0,328	0,222	0,215	0,234
	**KX355613.1**	EuRV	Euphorbia ringspot virus	1,003	1,014	0,993	0,985	0,562	1,237	1,114	1,351	1,489	0,774	0,779	0,639	1,107	0,885	1,064	0,908	10147	0,355	0,254	0,178	0,213
	**FM206346.1**	FreMV	Freesia mosaic virus	1,021	1,039	1,076	0,883	0,643	1,191	0,951	1,275	1,360	0,855	0,734	0,891	1,092	0,857	1,140	0,938	9489	0,311	0,260	0,175	0,254
	**AM039800.1**	FriVY	Fritillary virus Y	0,994	1,014	0,955	1,028	0,657	1,166	1,034	1,246	1,407	0,910	0,818	0,704	1,056	0,876	1,164	0,933	9656	0,316	0,256	0,186	0,242
	**EF427894.1**	GloSMV	Gloriosa stripe mosaic virus	1,002	1,036	1,077	0,884	0,672	1,136	0,996	1,314	1,446	0,833	0,730	0,794	1,002	0,931	1,129	0,969	9455	0,319	0,267	0,194	0,220
	**LC228573.1**	GoMV	Gomphocarpus mosaic virus	1,073	0,922	0,977	1,007	0,460	1,177	1,165	1,391	1,490	1,061	0,786	0,454	1,102	0,863	1,021	0,998	9998	0,315	0,259	0,188	0,238
	**AB818538.1**	HaMV	Habenaria mosaic virus	0,960	0,993	1,123	0,958	0,754	1,157	0,914	1,243	1,342	0,846	0,735	0,924	1,027	0,972	1,152	0,861	9499	0,319	0,251	0,199	0,231
	**HQ161081.1**	HarMV	Hardenbergia mosaic virus	1,048	0,915	0,989	1,036	0,556	1,153	1,061	1,400	1,442	1,128	0,776	0,417	1,082	0,845	1,124	0,963	9682	0,324	0,261	0,184	0,232
	**MH779476.1**	HenMV	Henbane mosaic virus	1,017	1,031	0,997	0,946	0,655	1,079	0,978	1,414	1,371	0,974	0,812	0,691	1,008	0,895	1,206	0,908	10140	0,313	0,250	0,213	0,224
	**JQ395040.1**	HipMV	Hippeastrum mosaic virus	1,035	0,956	0,999	1,004	0,589	1,241	1,003	1,242	1,386	0,917	0,786	0,776	1,115	0,855	1,156	0,900	9660	0,303	0,267	0,183	0,247
	**KY828925.2**	HyaMV	Hyacinth mosaic virus	1,003	1,016	1,099	0,906	0,648	1,213	0,909	1,294	1,432	0,803	0,838	0,772	1,061	0,894	1,091	0,969	9500	0,315	0,263	0,178	0,243
	**KU981084.1**	ImpFBV	Impatiens flower break potyvirus	1,061	0,974	1,025	0,927	0,700	1,160	0,966	1,257	1,313	0,907	0,842	0,788	1,040	0,913	1,128	0,953	9622	0,323	0,278	0,173	0,226
	**NC_018833.1**	IJMV	Iranian johnsongrass mosaic virus	1,015	0,974	1,078	0,936	0,645	1,238	1,058	1,241	1,336	0,851	0,752	0,859	1,082	0,901	1,035	0,952	9544	0,343	0,249	0,195	0,213
	**JF320812.1**	IrMMV	Iris mild mosaic virus	0,951	1,000	1,183	0,910	0,813	1,110	0,820	1,291	1,308	0,867	0,792	0,905	1,030	0,983	1,141	0,856	8651	0,316	0,270	0,193	0,221
	**KT692938.1**	IrSMV	Iris severe mosaic virus	1,061	0,970	1,062	0,884	0,596	1,203	1,019	1,409	1,366	0,941	0,738	0,679	1,083	0,855	1,089	0,964	10423	0,354	0,257	0,174	0,215
	**AB027007.1**	JapYMV	Japanese mild mosaic virus	1,017	0,976	1,057	0,953	0,596	1,193	1,084	1,338	1,432	0,877	0,739	0,693	1,053	0,929	1,043	0,961	9760	0,342	0,246	0,190	0,222
	**KT222674.1**	JasRV	Jasmine ringspot virus	1,015	0,991	1,046	0,952	0,658	1,137	1,035	1,288	1,319	0,990	0,751	0,780	1,092	0,871	1,101	0,933	9590	0,320	0,254	0,190	0,236
	**Z26920.1**	JoMV	Johnson grass mosaic virus	0,936	1,048	1,070	0,987	0,661	1,132	1,058	1,328	1,392	0,897	0,756	0,723	1,109	0,879	1,047	0,920	9779	0,343	0,235	0,197	0,224
	**KY385304.1**	KaMV	Kalanchoe mosaic virus	1,043	0,976	1,062	0,907	0,697	1,163	0,965	1,300	1,283	0,927	0,806	0,836	1,034	0,914	1,122	0,940	9462	0,329	0,254	0,198	0,219
	**JF838187.1**	KjMV	Keunjorong mosaic virus	1,078	0,919	1,000	0,987	0,581	1,255	0,993	1,264	1,306	0,989	0,888	0,715	1,105	0,841	1,097	0,961	9626	0,307	0,258	0,191	0,244
	**AB219545.1**	KoMV	Konjac mosaic virus	0,965	0,997	1,048	1,012	0,630	1,220	0,939	1,312	1,475	0,853	0,859	0,635	1,050	0,896	1,110	0,953	9544	0,320	0,247	0,190	0,243
	**AJ307057.1**	LeYSV	Leek yellow stripe potyvirus	0,968	0,996	1,081	0,983	0,745	1,127	0,945	1,244	1,332	0,941	0,771	0,804	1,060	0,911	1,137	0,910	10142	0,314	0,261	0,189	0,236
	**KP769852.1**	LeINV	Lettuce italian necrotic virus	0,964	1,012	1,087	0,970	0,650	1,152	1,007	1,348	1,449	0,842	0,780	0,687	1,085	0,938	1,044	0,904	9829	0,336	0,257	0,185	0,222
	**X97705.1**	LeMV	Lettuce mosaic virus	0,997	0,969	1,052	0,987	0,683	1,171	1,129	1,164	1,266	0,961	0,809	0,837	1,064	0,918	0,978	1,009	10080	0,329	0,223	0,212	0,237
	**AM048875.1**	LiMoV	Lily mottle virus	0,980	0,984	1,120	0,929	0,688	1,092	0,994	1,293	1,285	1,035	0,728	0,877	1,052	0,902	1,112	0,927	9648	0,295	0,230	0,227	0,248
	**MF543013.1**	LiYMV	Lily yellow mosaic virus	0,900	1,087	1,114	0,946	0,777	1,077	0,929	1,264	1,360	0,857	0,803	0,853	1,047	0,934	1,091	0,929	9811	0,309	0,241	0,204	0,246
	**EU847625.2**	LuMV	Lupine mosaic virus	0,958	1,037	1,072	0,964	0,718	1,094	0,982	1,355	1,466	0,906	0,772	0,558	1,009	0,908	1,096	1,013	10113	0,348	0,253	0,181	0,219
	**AM110758.1**	MDMV	Maize dwarf mosaic virus	1,015	0,982	1,046	0,956	0,660	1,167	1,029	1,314	1,341	0,928	0,771	0,754	1,075	0,894	1,100	0,918	9523	0,338	0,255	0,193	0,214
	**EF579955.1**	MWMV	Moroccan watermelon mosaic virus	1,043	0,941	0,956	1,042	0,618	1,229	1,067	1,208	1,317	0,954	0,823	0,763	1,116	0,858	1,128	0,899	9730	0,317	0,263	0,188	0,232
	**AM182028.1**	NarDV	Narcissus degeneration virus	1,050	1,002	0,978	0,947	0,708	1,132	1,035	1,230	1,238	0,921	0,790	0,913	1,085	0,909	1,145	0,880	9816	0,326	0,265	0,172	0,237
	**KC691259.1**	NarLSYV	Narcissus late season yellows virus	1,007	0,989	1,113	0,904	0,654	1,175	0,984	1,303	1,357	0,925	0,734	0,822	1,042	0,898	1,091	0,970	9687	0,319	0,244	0,203	0,235
	**AM158908.1**	NaYSV1	Narcissus yellow stripe virus 1	0,984	0,978	1,154	0,912	0,642	1,198	0,997	1,308	1,362	0,936	0,740	0,778	1,080	0,881	1,011	0,999	9650	0,328	0,240	0,201	0,230
	**AJ510223.1**	OnYDV	Onion yellow dwarf virus	0,992	1,036	1,116	0,877	0,765	1,084	0,985	1,278	1,256	0,876	0,737	0,958	1,098	0,939	1,047	0,884	10538	0,341	0,268	0,173	0,217
	**JQ807997.1**	OrMV	Ornithogalum mosaic virus	1,035	0,956	1,057	0,956	0,661	1,227	1,007	1,202	1,312	0,907	0,751	0,876	1,092	0,874	1,111	0,929	9445	0,319	0,265	0,185	0,231
	**GQ916624.1**	PanVY	Panax virus Y	1,010	0,968	1,044	0,988	0,736	1,137	1,015	1,219	1,292	0,925	0,852	0,775	1,064	0,944	1,037	0,939	9750	0,332	0,266	0,180	0,222
	**AB088221.1**	PapLDMV	Papaya leaf distortion mosaic virus	1,038	0,984	0,939	1,011	0,665	1,202	1,060	1,213	1,311	0,905	0,876	0,746	1,108	0,853	1,116	0,925	10153	0,334	0,270	0,174	0,222
	**X67673.1**	PapRV	Papaya ringspot Virus	1,069	0,940	0,975	0,996	0,625	1,169	1,061	1,253	1,261	1,023	0,858	0,739	1,136	0,869	1,071	0,916	10326	0,312	0,270	0,180	0,238
	**MF509898.1**	PMNV	Paris mosaic necrosis virus	1,023	1,010	0,972	0,978	0,546	1,235	1,046	1,344	1,503	0,846	0,804	0,613	1,078	0,847	1,141	0,951	9660	0,328	0,258	0,181	0,233
	**HQ122652.2**	PasFWV	Passion fruit woodiness virus	0,997	0,946	1,046	1,028	0,544	1,184	1,037	1,399	1,514	0,984	0,799	0,461	1,114	0,884	1,052	0,933	9680	0,324	0,261	0,181	0,234
	**D10930.1**	PeaSBMV	Pea seed-borne mosaic virus	1,007	0,956	1,034	1,014	0,578	1,266	0,991	1,292	1,511	0,849	0,753	0,653	1,066	0,889	1,142	0,926	9924	0,326	0,256	0,175	0,244
	**AF023848.1**	PeaMV	Peanut mottle virus	1,009	0,959	1,017	1,019	0,643	1,140	1,043	1,306	1,410	0,982	0,814	0,597	1,055	0,922	1,073	0,954	9708	0,326	0,254	0,181	0,240
	**U05771.1**	PeaSV	Peanut stripe virus	1,025	0,950	0,947	1,059	0,532	1,174	1,069	1,397	1,534	1,032	0,801	0,387	1,075	0,857	1,145	0,946	10056	0,325	0,259	0,177	0,239
	**KT633868.1**	PMaV	Pecan mosaic-associated virus	0,998	0,987	1,031	0,987	0,688	1,145	1,067	1,198	1,281	0,941	0,797	0,874	1,071	0,922	1,069	0,928	9310	0,309	0,242	0,208	0,241
	**AY642590.1**	PenMV	Pennisetum mosaic virus	0,958	0,994	1,075	1,011	0,726	1,202	0,934	1,265	1,359	0,870	0,819	0,726	1,082	0,882	1,113	0,906	9611	0,348	0,254	0,185	0,213
	**M96425.1**	PepMoV	Pepper mottle virus	1,010	1,015	0,986	0,981	0,673	1,105	1,042	1,292	1,369	0,965	0,843	0,657	1,079	0,885	1,094	0,953	9640	0,316	0,270	0,181	0,232
	**AM181350.1**	PepSMV	Pepper severe mosaic virus	1,041	0,946	0,969	1,034	0,624	1,173	0,983	1,308	1,409	0,987	0,830	0,601	1,074	0,877	1,195	0,898	9890	0,310	0,277	0,181	0,232
	**DQ645484.1**	PepVMV	Pepper veinal mottle virus	1,011	0,968	0,977	1,042	0,641	1,102	1,062	1,318	1,396	1,068	0,735	0,600	1,076	0,870	1,177	0,903	9792	0,314	0,267	0,189	0,231
	**AB541985.1**	PepYMV	Pepper yellow mosaic virus	1,022	1,027	1,004	0,938	0,610	1,138	1,016	1,357	1,396	0,913	0,812	0,718	1,084	0,879	1,133	0,913	9744	0,313	0,263	0,192	0,232
	**AJ516010.1**	PerTV	Peru tomato virus	1,050	0,942	0,945	1,044	0,603	1,193	1,021	1,288	1,434	1,009	0,847	0,522	1,069	0,843	1,166	0,966	9892	0,313	0,276	0,179	0,233
	**D13751.1**	PPV	Plum pox virus	1,029	0,956	1,028	0,984	0,553	1,187	1,122	1,296	1,410	0,951	0,768	0,700	1,087	0,898	1,034	0,964	9741	0,313	0,252	0,204	0,230
	**MG189944.1**	PokMV	Pokeweed mosaic virus	1,035	0,945	1,052	0,966	0,595	1,179	1,052	1,324	1,341	0,982	0,843	0,682	1,094	0,893	1,013	0,975	9516	0,317	0,253	0,206	0,223
	**Z21670.1**	PoVA	Potato virus A	0,958	0,992	1,000	1,069	0,693	1,037	1,070	1,345	1,434	1,089	0,795	0,453	1,011	0,893	1,106	1,008	9585	0,327	0,247	0,200	0,225
	**AJ243766.2**	PoVV	Potato virus V	1,065	0,928	0,966	1,023	0,582	1,188	0,929	1,390	1,410	1,005	0,878	0,561	1,049	0,883	1,219	0,892	9848	0,309	0,262	0,190	0,239
	**KR528584.1**	PoVY	Potato virus Y	1,013	1,001	0,957	1,014	0,681	1,080	1,068	1,281	1,367	1,028	0,804	0,629	1,072	0,884	1,127	0,941	9678	0,313	0,272	0,181	0,234
	**KY562565.1**	SaLV	Saffron latent virus	1,016	0,971	0,984	1,023	0,633	1,173	1,042	1,310	1,468	0,945	0,817	0,503	1,067	0,880	1,108	0,965	9693	0,337	0,268	0,166	0,228
	**AJ316084.1**	ScaMV	Scallion mosaic virus	0,942	0,979	1,107	1,017	0,737	1,208	0,933	1,207	1,369	0,857	0,820	0,778	1,056	0,926	1,071	0,939	9324	0,329	0,248	0,193	0,230
	**AJ865076.1**	ShYSV	Shallot yellow stripe virus	0,950	1,024	1,129	0,949	0,764	1,154	0,955	1,215	1,354	0,816	0,696	0,911	1,074	0,929	1,094	0,898	10429	0,340	0,260	0,175	0,225
	**AJ310197.1**	SorMV	Sorghum mosaic virus	0,980	1,012	1,030	0,994	0,718	1,197	1,020	1,214	1,303	0,884	0,839	0,773	1,111	0,841	1,068	0,949	9624	0,351	0,256	0,184	0,209
	**NC_002634.1**	SoyMV	Soybean mosaic virus	1,019	0,917	0,998	1,068	0,566	1,218	0,991	1,341	1,507	0,991	0,896	0,416	1,069	0,878	1,091	0,974	9588	0,318	0,263	0,180	0,240
	**KY623505.1**	SuWMV	Sudan watermelon mosaic virus	1,014	0,979	0,998	1,006	0,656	1,167	1,071	1,221	1,297	0,969	0,838	0,774	1,106	0,875	1,059	0,943	10232	0,314	0,254	0,196	0,236
	**AM110759.1**	SuMV	Sugarcane mosaic virus	0,977	1,007	1,055	0,978	0,721	1,149	1,001	1,269	1,345	0,908	0,795	0,754	1,028	0,908	1,105	0,962	9596	0,338	0,244	0,202	0,216
	**GU181199.1**	SuCMOV	Sunflower chlorotic mottle virus	1,047	0,950	0,981	1,007	0,559	1,223	1,130	1,273	1,423	0,892	0,731	0,730	1,107	0,897	1,096	0,887	9965	0,326	0,267	0,185	0,222
	**JQ350738.1**	SunMMV	Sunflower mild mosaic virus	1,000	1,040	1,007	0,952	0,670	1,142	1,071	1,263	1,344	0,853	0,787	0,847	1,051	0,920	1,101	0,923	9718	0,329	0,244	0,199	0,228
	**KX856009.1**	SunRBV	Sunflower ring blotch virus	1,076	0,939	1,057	0,912	0,496	1,267	1,139	1,311	1,481	0,836	0,650	0,785	1,079	0,910	1,052	0,944	9555	0,327	0,265	0,190	0,218
	**KU511268.1**	SPFMV	Sweet potato feathery mottle virus	1,015	1,012	1,013	0,956	0,648	1,102	0,988	1,386	1,357	0,996	0,945	0,551	1,080	0,874	1,040	0,996	10794	0,324	0,257	0,187	0,232
	**KC443039.1**	SwPLV	Sweet potato latent virus	0,958	1,090	0,992	0,967	0,723	1,071	0,951	1,340	1,351	0,910	0,868	0,724	1,073	0,873	1,170	0,901	10081	0,320	0,254	0,190	0,235
	**N613807.1**	SwPV2	Sweet potato virus 2	1,038	0,964	1,004	0,984	0,626	1,143	1,021	1,363	1,315	1,043	0,902	0,572	1,121	0,859	1,047	0,948	10731	0,332	0,259	0,179	0,231
	**GU207957.1**	SwPVC	Sweet potato virus C	1,004	0,973	1,049	0,985	0,660	1,163	0,956	1,311	1,342	0,992	0,872	0,657	1,084	0,868	1,085	0,963	10820	0,315	0,255	0,191	0,238
	**JN613806.1**	SwPVG	Sweet potato virus G	1,016	0,946	1,031	1,012	0,646	1,197	0,965	1,320	1,305	1,043	0,932	0,573	1,117	0,831	1,048	0,979	10800	0,331	0,251	0,185	0,233
	**KM523548.1**	TaLMV	Tamarillo leaf malformation virus	1,026	0,924	1,053	1,013	0,608	1,111	1,049	1,391	1,437	1,061	0,829	0,433	1,090	0,923	1,003	0,963	9720	0,322	0,274	0,182	0,222
	**DQ851493.1**	TeMV	Telosma mosaic virus	1,034	0,919	0,982	1,060	0,540	1,204	1,058	1,358	1,502	1,013	0,857	0,397	1,097	0,867	1,068	0,967	9689	0,322	0,268	0,178	0,232
	**AJ851866.1**	ThFMV	Thunberg fritillary virus	1,001	0,972	1,127	0,932	0,753	1,159	0,889	1,251	1,272	0,966	0,778	0,837	1,058	0,892	1,119	0,946	9723	0,323	0,258	0,183	0,236
	**M11458.1**	TobEV	Tobacco etch virus	1,012	0,963	1,010	1,016	0,627	1,179	0,977	1,316	1,417	1,011	0,823	0,586	1,047	0,850	1,154	0,975	9494	0,313	0,255	0,191	0,240
	**KT834407.1**	TobMV	Tobacco mosqueado virus	1,006	0,938	0,978	1,083	0,639	1,171	1,042	1,273	1,447	0,992	0,839	0,518	1,031	0,893	1,118	0,979	9885	0,316	0,264	0,193	0,228
	**EF219408.1**	TobVBMV	Tobacco vein banding mosaic virus	1,017	0,987	0,990	1,001	0,639	1,158	1,066	1,265	1,408	0,927	0,820	0,668	1,056	0,897	1,088	0,968	9570	0,317	0,265	0,190	0,228
	**X04083.1**	TVMV	Tobacco vein mottling virus	1,026	1,000	0,898	1,048	0,629	1,173	1,076	1,259	1,424	0,961	0,791	0,627	1,032	0,836	1,231	0,950	9475	0,320	0,261	0,191	0,229
	**JQ314463.1**	ToNSV	Tomato necrotic stunt virus	1,032	0,921	0,945	1,095	0,691	1,085	1,048	1,257	1,298	1,128	0,979	0,486	1,086	0,901	1,031	0,983	10057	0,302	0,276	0,186	0,237
	**AF169561.2**	TurMV	Turnip mosaic virus	0,956	0,923	1,022	1,109	0,657	1,138	1,142	1,198	1,315	0,991	0,763	0,804	1,098	0,982	1,047	0,847	9835	0,319	0,225	0,213	0,244
	**KF906523.1**	VaDMV	Vanilla distortion mosaic virus	0,947	1,049	1,096	0,935	0,663	1,195	1,123	1,155	1,303	0,893	0,732	0,952	1,071	0,871	1,023	0,998	9553	0,317	0,208	0,231	0,243
	**EU564817.1**	VerVY	Verbena virus Y	1,053	0,993	1,028	0,908	0,590	1,190	1,107	1,284	1,403	0,839	0,720	0,836	1,061	0,925	1,071	0,940	9742	0,326	0,261	0,189	0,224
	**AY437609.1**	WaMV	Watermelon mosaic virus	0,988	0,935	1,044	1,058	0,664	1,247	0,952	1,217	1,431	0,928	0,819	0,626	1,073	0,865	1,128	0,956	10035	0,323	0,265	0,175	0,237
	**KY623506.1**	WiMVBV	Wild melon vein banding virus	1,028	0,932	1,054	0,991	0,649	1,190	1,031	1,242	1,247	1,057	0,754	0,811	1,133	0,841	1,099	0,911	10152	0,316	0,252	0,195	0,237
	**LC159494.1**	WiOSV	Wild onion symptomless virus	0,976	0,990	1,085	0,973	0,681	1,165	0,974	1,305	1,369	0,873	0,747	0,821	1,052	0,951	1,118	0,874	9369	0,330	0,241	0,197	0,232
	**AJ437279.1**	WiPMV	Wild potato mosaic virus	1,032	0,939	0,966	1,051	0,657	1,182	0,990	1,257	1,393	1,013	0,774	0,644	1,035	0,869	1,232	0,921	9878	0,313	0,265	0,184	0,238
	**DQ851495.1**	WTMV	Wild tomato mosaic	1,036	1,004	0,976	0,965	0,608	1,123	1,024	1,383	1,410	0,941	0,847	0,617	1,094	0,893	1,128	0,894	9659	0,318	0,274	0,182	0,225
	**AY656816.1**	WisVMV	Wisteria vein mosaic	1,031	0,947	0,930	1,071	0,585	1,163	1,038	1,398	1,501	1,015	0,898	0,304	1,087	0,873	1,133	0,927	9694	0,338	0,271	0,165	0,226
	**JN190431.1**	YBMV	Yam bean mosaic virus	1,005	0,990	0,937	1,050	0,594	1,135	1,038	1,391	1,515	1,003	0,842	0,397	1,059	0,864	1,161	0,948	9648	0,331	0,258	0,174	0,238
	**JX470965.1**	YMMV	Yam mild mosaic virus	1,007	0,966	1,104	0,945	0,616	1,234	0,986	1,332	1,421	0,899	0,686	0,728	1,089	0,864	1,117	0,922	9538	0,340	0,257	0,183	0,220
	**U42596.1**	YMV	Yam mosaic virus	1,040	0,955	1,049	0,958	0,614	1,141	1,035	1,353	1,390	0,985	0,802	0,622	1,092	0,914	1,041	0,940	9608	0,326	0,266	0,175	0,234
	**AY626825.4**	ZMMV	Zantedeschia mild mosaic virus	1,065	0,977	0,970	0,965	0,642	1,170	0,991	1,306	1,361	0,974	0,885	0,645	0,976	0,874	1,151	1,034	9973	0,313	0,248	0,205	0,234
	**KU355553.1**	ZSSV	Zucchini shoestring virus	1,065	0,932	0,985	0,998	0,636	1,180	1,089	1,221	1,308	0,967	0,804	0,780	1,062	0,921	1,080	0,938	10308	0,317	0,259	0,191	0,234
	**KC345605.1**	ZTMV	Zucchini tigre mosaic virus	1,120	0,938	0,977	0,927	0,610	1,133	1,119	1,280	1,208	1,060	0,759	0,849	1,108	0,887	1,093	0,907	10340	0,315	0,262	0,189	0,235
	**AF127929.2**	ZYMV	Zucchini yellow mosaic virus	1,033	0,970	0,981	1,005	0,593	1,262	0,999	1,248	1,336	0,949	0,794	0,784	1,121	0,797	1,195	0,902	9591	0,310	0,257	0,195	0,238
**Roymovirus**	**MH697610.1**	PYRAV	dendron yellow ringspot associated	1,111	0,781	1,088	1,017	0,583	1,328	0,927	1,250	1,290	0,995	0,890	0,721	1,062	0,940	1,054	0,941	10457	0,311	0,254	0,195	0,240
	**JF280796.2**	RoYMV	Rose yellow mosaic virus	1,012	0,956	1,041	1,000	0,561	1,218	1,039	1,280	1,336	0,979	0,815	0,753	1,196	0,829	1,058	0,894	9508	0,300	0,265	0,199	0,236
**Rymovirus**	**AY623626.1**	AgMV	Agropyron mosaic virus	1,022	0,996	1,040	0,937	0,580	1,194	1,081	1,305	1,340	0,962	0,713	0,837	1,097	0,836	1,129	0,917	9540	0,317	0,242	0,214	0,227
	**AY623627.1**	HoMV	Hordeum mosaic virus	0,975	0,989	1,121	0,933	0,671	1,200	0,947	1,316	1,345	0,915	0,731	0,848	1,045	0,893	1,137	0,917	9463	0,325	0,232	0,215	0,228
	**Y09854.1**	RyMV	Reygrass mosaic virus	0,967	1,095	1,069	0,889	0,701	1,114	0,963	1,303	1,295	0,891	0,710	0,994	1,058	0,870	1,211	0,858	9535	0,307	0,231	0,220	0,243
**Tritimovirus**	**Z48506.1**	BrSMV	Brome streak mosaic virus	1,018	0,974	1,032	0,977	0,660	1,177	0,938	1,282	1,285	0,971	0,750	0,902	1,059	0,890	1,227	0,847	9672	0,297	0,237	0,210	0,256
	**AY377938.1**	ONMV	Oat necrotic mottle virus	0,994	1,001	1,074	0,945	0,592	1,133	1,017	1,369	1,434	0,911	0,727	0,772	1,023	0,949	1,124	0,916	9346	0,309	0,233	0,210	0,249
	**KF260962.1**	TOMV	Tall oatgrass mosaic virus	0,930	1,089	0,941	1,048	0,860	1,009	0,935	1,216	1,240	0,935	0,969	0,783	1,042	0,928	1,161	0,896	9359	0,318	0,241	0,188	0,252
	**KF984546.1**	YOGMV	Yellow oat-grass mosaic virus	0,974	1,033	1,019	0,989	0,744	1,102	0,925	1,288	1,301	0,939	0,816	0,824	1,023	0,916	1,191	0,896	9292	0,318	0,227	0,201	0,254
	**EF608612.1**	WEMV	Wheat eqlid mosaic virus	1,026	1,017	1,037	0,918	0,678	1,073	0,999	1,332	1,303	0,935	0,812	0,838	1,060	0,955	1,109	0,884	9635	0,305	0,257	0,197	0,241
	**AF057533.1**	WSMV	Wheat streak mosaic virus	0,974	1,021	1,076	0,952	0,598	1,220	0,985	1,320	1,436	0,856	0,697	0,823	1,064	0,880	1,167	0,897	9384	0,316	0,237	0,202	0,245
**Unkonwn**	**KY649478.1**	LWBaV	ngan witches broom-associated vir	1,080	0,955	0,986	0,974	0,575	1,179	1,061	1,217	1,318	1,007	0,984	0,658	1,130	0,845	0,961	1,059	9428	0,274	0,277	0,198	0,251
	**KY612317.1**	CRCSV	Reed chlorotic stripe virus	0,989	0,975	1,073	0,959	0,672	1,186	1,045	1,222	1,300	0,943	0,785	0,880	1,001	0,921	1,091	0,976	9426	0,310	0,216	0,247	0,228

	AA	AT	AC	AG	TA	TT	TC	TG	CA	CT	CC	CG	GA	GT	GC	GG
Average	1,020	0,972	1,023	0,984	0,632	1,176	1,018	1,295	1,370	0,945	0,811	0,707	1,078	0,886	1,106	0,932
Standard deviation	0,041	0,044	0,059	0,057	0,066	0,053	0,063	0,061	0,075	0,068	0,076	0,146	0,042	0,039	0,060	0,044

	Significance	Values
Underrepresented < 0.78	*	0.78-0.70
	**	0.50-0.70
	***	<0.50
Overrepresented > 1.23	*	1.23-1.3
	**	1.30-1.5
	***	>1.50

Unclassified potyvirus
